# Impact of different protein sources on acute muscle protein synthesis following resistance training: a systematic review with standard and dose–response network meta-analyses

**DOI:** 10.1080/15502783.2026.2740224

**Published:** 2026-10-12

**Authors:** Kevin Bischof, Anna Maria Kripp, Maria Petropoulou, Brendon Stubbs, Daniel König

**Affiliations:** a Centre for Sports Science and Department of Sports Science, Section for Nutrition, Exercise and Health, University Sports, University of Vienna, Vienna, Austria; b Faculty of Life Sciences, Department for Nutrition, Section for Nutrition, Exercise and Health, University of Vienna, Vienna, Austria; c Institute of Medical Biometry and Statistics, Faculty of Medicine and Medical Center, University of Freiburg, Freiburg, Germany; d Psychological Medicine, Institute of Psychiatry, Psychology and Neuroscience (IoPPN), King’s College London, London, UK; e Vienna Doctoral School of Pharmaceutical, Nutritional and Sport Sciences, University of Vienna, Vienna, Austria

**Keywords:** Muscle protein synthesis, fractional synthetic rate, resistance training, protein source

## Abstract

**Background:**

The combination of protein intake and resistance training (RT) plays a pivotal role in stimulating muscle protein synthesis (MPS), yet the impact of different protein sources remains an area of active investigation. Therefore, a systematic review incorporating standard and dose‒response network meta-analyses was performed.

**Methods:**

Systematic literature search was conducted in PubMed, EBSCOhost (including MEDLINE, SPORTDiscus, CINAHL), and Scopus. Eligible studies included healthy adults (≥18 years) who were undergoing acute resistance exercise followed by a single protein bolus (any type or dose, except blends or enriched with anabolic agents). Muscle biopsies were required before and after RT/protein intake without additional food in between. Trials measuring hourly MPS rates via tracer techniques were included (*n* = 51).

**Results:**

In the standard network meta-analysis (NMA), native whey (P-score = 0.81, MD = 0.05% [0.00; 0.10]), pea protein (0.77, MD = 0.05% [−0.02; 0.11]), whole egg protein (0.72, MD = 0.04% [−0.01; 0.09]), mycoprotein (0.66, MD = 0.03% [−0.02; 0.08]), and whey protein (0.58, MD = 0.02% [0.01; 0.04]) ranked highest for estimated efficacy in stimulating post-RT hourly MPS (in descending order) (only whey protein showed a statistically significant effect) with low to moderate certainty of evidence. Dose‒response NMA revealed that certain animal- (native whey and whole egg protein) and plant- (pea and mycoprotein) based protein sources to show the highest predicted MPS responses with doses of 18–25 g.

**Conclusions:**

Animal- (native whey, whey, whole egg) and plant-based (pea, mycoprotein) proteins ranked similarly for estimated acute MPS stimulation when combined with RT; however, only whey protein showed a statistically significant effect, and protein sources represented by few studies should be interpreted with caution.

## 1. Introduction

Resistance training (RT) in conjunction with protein intake is a well-established strategy to stimulate muscle protein synthesis (MPS), depicting an indicator of protein turnover and remodeling of muscular tissue. Enhancing MPS is critical for the accrual of skeletal muscle mass and plays a pivotal role in optimizing athletic performance. Moreover, it represents a key strategy in older adults to improve mobility and counteract sarcopenia—the progressive, age-related loss of muscle mass and function [1]. A commonly used method to gauge the muscle protein fractional synthesis rate (FSR) in vivo represents the precursor–product method, which involves tracing stable isotope-labeled amino acids during a short post-exercise and/or nutrition window, with later advancements enabling the assessment of specific muscle protein fractions such as myofibrillar or mitochondrial FSR [2].

As previously outlined in a meta-analysis, MPS rates are influenced by factors such as age, training status, timing of measurement, and RT variables (e.g. number of sets and repetitions, intensity) [3], necessitating the consideration of inherent heterogeneity when interpreting findings. Regarding training status, untrained individuals' MPS appears to peak later but remains increased for a longer duration following RT, resulting in a greater overall post-exercise MPS response compared to resistance-trained athletes. Putatively, the significantly higher muscle fiber damage after an initial bout of resistance exercise keeps the MPS elevated for a longer duration, whereas MPS rates following repeated bouts of RT continuously decline [4]. The timing of MPS assessment—determined by the interval between muscle biopsies—represents a critical methodological factor, as the true MPS response occurs within a specific temporal window. If the second biopsy is taken too early or too late—before MPS returns to baseline or after it has already subsided—the resulting FSR may underestimate or misrepresent the true response [2].

RT, as one of the most potent contributor to muscle size accrual, can stimulate MPS at a rate of approximately 0.03% per hour [3], which can increase to around 0.05% per hour when combined with additional protein in a dose-dependent manner [5]. Accordingly, a study demonstrated a dose-dependent increase in MPS rates following the ingestion of whole egg protein drinks containing 5 g, 10 g, 20 g, or 40 g of protein combined with RT [6]. Nevertheless, MPS rates did not differ significantly between the 20 g and 40 g doses, nor between the 5 g and 10 g doses, indicating a non-linear dose–response relationship. As indicated by a review of the literature, an optimal protein intake of 30–45 g per meal [7] or 0.4–0.6 g/kg bodyweight—corresponding to approximately 1.6–2.2 g/kg/day—is suggested to maximize muscle mass and strength [8]; however, higher bolus intakes may further enhance this effect, albeit with increased amino acid oxidation as a potential drawback [9,10].

In recent decades, the socio-nutritional behavior of (younger) individuals has shifted markedly from predominantly omnivorous, meat-containing diets towards vegetarian and even vegan dietary patterns, the latter of which are associated with various health benefits [11] and a climate-conscious reduction in greenhouse gas emissions [12]. Plant-based diets and plant-derived protein sources have historically been regarded as less effective at stimulating muscle protein metabolism compared to animal-based proteins. The lower anabolic potential of plant-based proteins may stem from reduced digestibility (mainly having a protein digestibility-corrected amino acid score < 0.9 [13]), greater splanchnic amino acid extraction and urea synthesis, as well as lower levels of essential amino acids—particularly leucine—compared to animal-based proteins [14]. Owing to leucine's distinctive ability to activate mammalian target of rapamycin (mTOR) in early in-vitro trials, the “leucine trigger” hypothesis suggests that postprandial MPS is, to some extent, proportional to plasma leucine concentrations, with muscle exposure to leucine playing a key regulatory role [15]. Recent systematic [16,17] reviews [18] without meta-analyses have outlined the increased needs of essential amino acids, especially of leucine and particularly in older adults, to maximize MPS. To achieve comparable anabolic effects with plant-based proteins characterized by a rather low essential amino acid content, it has been suggested that increasing the total protein intake may serve as an effective compensatory strategy [19]. Furthermore, animal-derived collagen protein has shown anabolic effects via increased muscle mass [20] although its low leucine profile. It has been proposed that certain bioactive di-, tri-, and oligopeptides—though still in the early stages of research—may elicit comparable anabolic effects [21].

Therefore, the aim of this systematic review, standard and dose–response NMA, is to comprehensively evaluate whether RT combined with leucine and essential amino acid abundant proteins increases MPS to a higher extent than RT combined with proteins containing lower levels of these amino acids (e.g. plant derived protein) in healthy adults and to determine the optimal intake doses for each protein type.

## 2. Methods

This systematic review and network meta-analysis was prospectively registered at Open Science Framework (OSF: https://doi.org/10.17605/OSF.IO/NYH65). The review was conducted in accordance with the PRISMA extension statement for network meta-analyses of health care interventions [22].

### 2.1. Eligibility criteria

Eligible participants were required to be healthy males or females, aged 18 years or older, and free from any chronic or severe illnesses (Table 1). Regarding study design, acute resistance exercise bouts combined with a subsequent bolus intake of any type of protein—irrespective of its processing state (e.g. isolate, hydrolysate, concentrate, or whole food sources such as cheese or milk but no whole meal) and dosage—were considered suitable. Muscle biopsies had to be carried out ahead of and in proximity to RT and several hours after RT plus protein ingestion so that no additional food could be consumed between the two biopsies. Thus, MPS rates derived from a whole day were not considered for inclusion. Study arms were excluded if the protein supplements consisted of blends or were enriched with additional amino acids or other anabolic agents, such as creatine, to avoid confounding, as these co-ingredients independently stimulate MPS and would likely prevent attribution of observed effects to the protein source and dose alone. In contrast, protein supplements added with carbohydrates were considered eligible, as there appears to be no beneficial effect on MPS [23]. Given the network meta-analysis framework, comparator interventions could include not only placebo or control supplements (e.g. maltodextrin, water) but also any eligible form of protein as defined above. In addition, data from single arms derived from single- or multi arm trials were eligible. Randomized controlled as well as crossover trials were to be included whereas observational, single case, qualitative, animal, *in vitro,* and *ex vivo* studies were excluded. The primary outcome was the hourly MPS rate [%] (corresponds to the fractional synthetic rate (FSR), the rate at which a precursor compound is incorporated into a product per unit of product mass [24]), assessed via stable isotope-labeled amino acid tracing in combination with muscle biopsy sampling. MPS measures included sarcoplasmic, myofibrillar, collagenous/connective tissue, and mitochondrial protein synthesis rates to comprehensively represent muscle protein turnover. Deviations from the original study protocol were necessary: collagen protein synthesis had to be excluded as a standalone analysis due to an insufficient number of studies, and daily protein synthesis rates (%) derived from deuterated water methodologies [25] were discarded owing to considerable heterogeneity across study designs. Thus, studies including multiple RT sessions were not considered eligible.

**Table 1.** PICOS criteria for trials' eligibility.

Component	Inclusion	Exclusion
Population	Healthy adults aged 18 years or older	Chronic or severe illness
Intervention	Acute resistance exercise bouts combined with subsequent intake of any type of protein	Whole meal, anabolic agents such as creatine, added amino acids
Comparator	Acute resistance exercise bouts combined with subsequent intake of any type of protein or placebo (water, carbohydrates)	Whole meal, anabolic agents such as creatine, added amino acids
Outcomes	Muscle derived sarcoplasmic, myofibrillar, collagenous/connective tissue, and mitochondrial protein synthesis rates	
Study design	Randomized controlled & crossover trials	observational, single case, qualitative, animal, *in vitro,* and *ex vivo* studies

### 2.2. Information sources and search strategy

A comprehensive literature search was carried out in PubMed, EBSCOhost (including MEDLINE, SPORTDiscus, CINAHL), and Scopus on 20.02.2025 with the following search string: (myofibrillar or muscle or muscular or connective tissue or collagen*) and protein and (synthe* or synthe* rate* or accretion or anaboli*) and (exercise or training) not (meta-analysis or systematic review or literature review or animal* or rodent* or mice or rat*). Filters for PubMed included humans, randomized controlled, and clinical trials; for Scopus, article and conference paper, article title, abstract and keywords were ticked, and “AND NOT” instead of “NOT” was used as a Boolean operator. An individual search has also been applied in Google Scholar and reference lists of suitable articles. Grey literature (e.g. posters, theses, congress abstracts) was eligible. No restrictions or limitations, such as publication date or language have been imposed. The result of the search & screen process are depicted in Figure 1.

**Figure 1.**
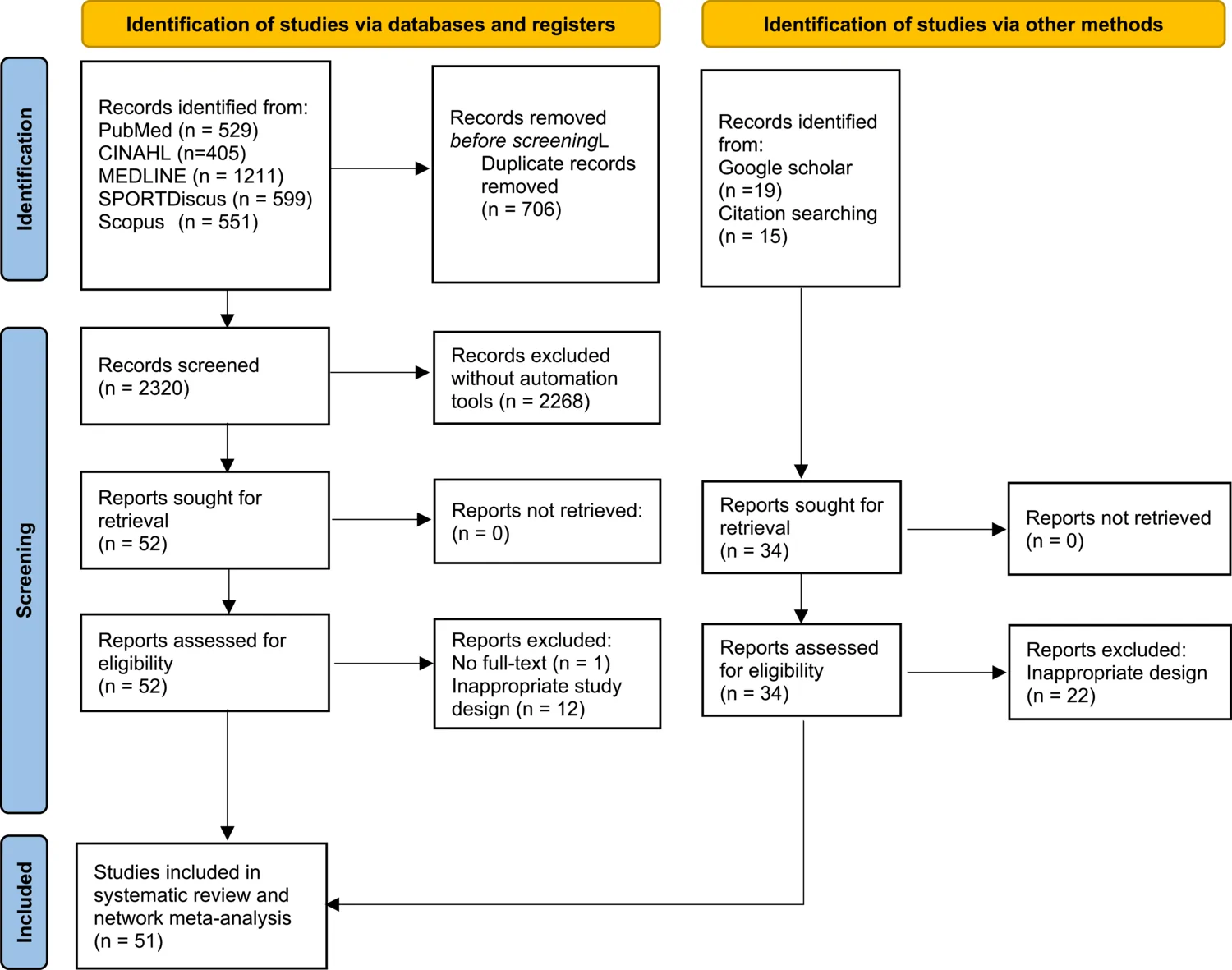
PRISMA flow chart.

### 2.3. Study selection, data collection process, and items

Two authors (A.M.K., K.B.) have independently screened the search results with the free online Rayyan tool (https://www.rayyan.ai/) to ensure impartiality and accuracy. Disagreements were resolved by discussion or consultation with a third and fourth author (M.P., D.K.) if necessary. Data from the included studies were primarily extracted by K.B. directly from the published articles or obtained from the study authors upon request. Data were sought for the following items: publication year, age, sample size, study type, gender, training status, protein + dosage, type of MPS, measurement duration, amino acid tracer, and mean ± standard deviation (SD) of MPS [%]. A list of included studies with corresponding items is shown in Table S1. If only g/kg bodyweight for protein source was given, the total amount was calculated and averaged based on participant baseline characteristics. In the case of data inaccessibility, WebPlotDigitizer v4 (https://automeris.io/) was used to extract means ± SDs from the figures. If only the standard error (SE) was reported, the SD was calculated using the formula SD = SE × √N [26].

### 2.4. Risk of bias and certainty of evidence assessment

Two authors (A.M.K., K.B.) independently assessed the risk of bias in the included studies using the Cochrane Risk of Bias tool for randomized trials (RoB 2) [27]; any discrepancies were resolved through consensus. The robvis tool [28] allowed for an exact illustration of the results. According to RoB 2, bias may arise from the randomization process due to deviations from the intended interventions due to missing outcome data, the measurement of the outcome, and the selection of the reported result, giving an overall risk of bias being either low, of some concerns or high. The Grading of Recommendations Assessment, Development, and Evaluation (GRADE) has been used to appraise the certainty of evidence in a network meta-analysis (done by K.B.) [29]. Starting with “high”, reasons for downgrading to “moderate”, “low” or “very low” certainty are risk of bias, indirectness, inconsistency, and publication bias. According to advances in the GRADE approach, rating imprecision is unnecessary when rating both direct and indirect estimates to inform the NMA estimates rating. We assessed the certainty of indirect estimates by referencing the direct comparisons that formed the most dominant first-order loop—defined as the triangular set of nodes contributing most substantially to a given indirect estimate—using the lower of the two direct ratings. In cases of serious intransitivity, the certainty of the indirect estimates was downgraded. To evaluate the overall certainty of the network estimates, we subsequently compared the ratings of corresponding direct and indirect evidence [29].

### 2.5. Statistical analysis

Continuous outcome data in analyses were summarized as the mean differences and their 95% confidence intervals (MD, 95% CI). We applied several models, including the frequentist graph-theoretical NMA [30] as well as a series of dose–response network meta-analysis (DR-NMA) models, to account for different functional forms of the dose–response relationship [31]. The standard NMA is considered the primary analysis; DR-NMA was conducted as a secondary analysis to characterize dose‒response relationships.

Specifically, we assessed the following DR-NMA models:

- Linear, exponential, and quadratic polynomial models
- First-order fractional polynomial (FP1) transformations with power p from the set {−2, −1, −0.5, 0, 0.5, 1, 2, 3}
- Restricted cubic splines (RCS) with knots placed at 10%, 50%, 90% percentiles (RCS) and 25%, 50%, 100% percentiles (RCS2)

Based on the model comparison analysis, FP1 (*p* = 0.5) demonstrated the best fit, with the lowest Q/df ratio (9.79) (detailed information in Supplementary File 1).

A random-effects model was undertaken, as studies differ both clinically and methodologically (i.e. between-study variability). The between-study variability (i.e. heterogeneity) of the intervention effects within each treatment comparison was assessed by I^2^ statistics, and the magnitude of the between-study variance was estimated using the generalized DerSimonian and Laird estimator ( ${\hat{\tau }}^{2})$ and the Q-profile approach.

Model adequacy relative to its complexity was evaluated based on the Q/(degrees of freedom (df)) ratio, which quantifies how well each model explains heterogeneity while avoiding unnecessary complexity. For each NMA, we evaluated the transitivity assumption, which implies that the distribution of potential treatment effect modifiers is balanced across the available direct comparisons [32]. We used age, sex, risk of bias, study design (non-/crossover trial), and training status (no training regimen/low physical activity/merely “healthy”, no RT history/moderately trained/recreationally active, RT trained—a minimum of two RTs/week) as potential intervention effect modifiers. Amino acid profile and resistance training (RT) characteristics (e.g. volume, intensity) were considered as potential modifiers but were not formally assessed as effect modifiers due to the lack of standardized reporting across the included studies, which precluded meaningful subgroup analyses. We evaluated each network for inconsistency using the random-effects design-by-treatment interaction model [33–35]. To assess local inconsistency, we applied the node-splitting method by splitting the direct and indirect evidence [36], and net heat plots [37].

P-scores (as part of the applied frequentist framework) were calculated to receive a relative ranking of protein sources, where higher P-score score values represent a greater probability of ranking as the most efficacious intervention for stimulating MPS relative to all other compared interventions (ranging from 0.0 exhibiting the worst intervention, to 1.0 (=100% certainty), reflecting the best intervention, both with no uncertainty) [35]. When the number of studies was more than ten, a comparison-adjusted funnel plot was drawn to assess publication bias and small study-effects. Subgroup analyses included age (<40>), sex (male/female/both) and type of protein synthesis (sarcoplasmic, myofibrillar, collagenous/connective tissue, and mitochondrial protein synthesis obtained by muscle biopsy). A sensitivity analysis was conducted to evaluate the extent to which MPS outcomes were influenced by a high risk of bias and the presence of crossover trials. All analyses were conducted using the R package “netmeta” [38], for NMA and “netdose” [31], for DR-NMA model.

## 3. Results

3026 articles were identified through the application of the search string (PubMed [529], EBSCOhost [MEDLINE [1211], SPORTDiscus [599], CINAHL [405]), and Scopus [551] (Figure 1). After the removal of 706 duplicates, 2269 records were excluded, primarily because of a lack of investigation into MPS rates, the inclusion of preclinical animal studies, review articles, or studies focusing on structural muscle adaptations such as cross-sectional area. Combining systematic and non-database search strategy, 51 studies were considered for the systematic review and network meta-analysis. Further details are found in Supplementary File 1.

After correcting for crossover designs, 1128 participants (906 men; 222 women) were investigated. Eleven trials employed a crossover design, one of them including three groups—two of which followed a crossover protocol, while the third was conducted as a parallel group. The publication year ranged from 2007 to 2024 with 21 articles published in the last five years. MPS rates were obtained from a period of 2–7 h, and total protein intake ranged from 5 to around 69 g (originally given as g/kg bodyweight). 34 and 19 studies included young (18–40 years) and older (41–80 years) individuals, respectively (with two studies including both). Of the 117 analyzed study/treatment arms, the collagenous, mixed, and myofibrillar MPS rates were obtained from 12, 28, and 77 arms, respectively. Protein sources demonstrated beef (4), salmon (1), milk (1), quark (2), cheese (1), chlorella (1), spirulina (1), egg white (1), whole eggs (1), and casein (11); protein from soy (4), fava bean (1), meat (1), mealworm (1), milk (12), pea (2), potato (1), and whole eggs (4); and collagen (5), whey (37), and native whey (1), as well as mycoprotein (4) and its concentrate (1) (number of study arms in parentheses; the rest reflected placebo). Biopsies were exclusively taken from the musculus vastus lateralis. Leucine, alanine and, most of the time, phenylalanine were used as labeled amino acid tracers.

### 3.1. Standard and dose–response network meta-analysis

The network geometry of studies examining 19 different protein sources in relation to acute MPS is shown in Figure 2. A summary of the model performance across all standard and DR-NMA models is provided in Table S2. The FP1 model with power *p* = 0.5 demonstrated the lowest Q/df ratio (9.79), indicating the best balance between model fit and parsimony. Accordingly, FP1 with power 0.5 was selected for reporting the DR-NMA results. In contrast, the standard NMA model exhibited a higher Q/df ratio (13.65), reflecting poorer model adequacy compared to lower-order FP1s, linear, exponential, and RCS dose–response models. Furthermore, the heterogeneity estimate for the standard NMA was substantially higher ( ${\hat{\tau }}^{2}$ = 0.0213, I^2^ = 92.7%) than that of the FP1 (*p* = 0.5) model ( ${\hat{\tau }}^{2}$ = 0.0049, I^2^ = 89.8%).

**Figure 2.**
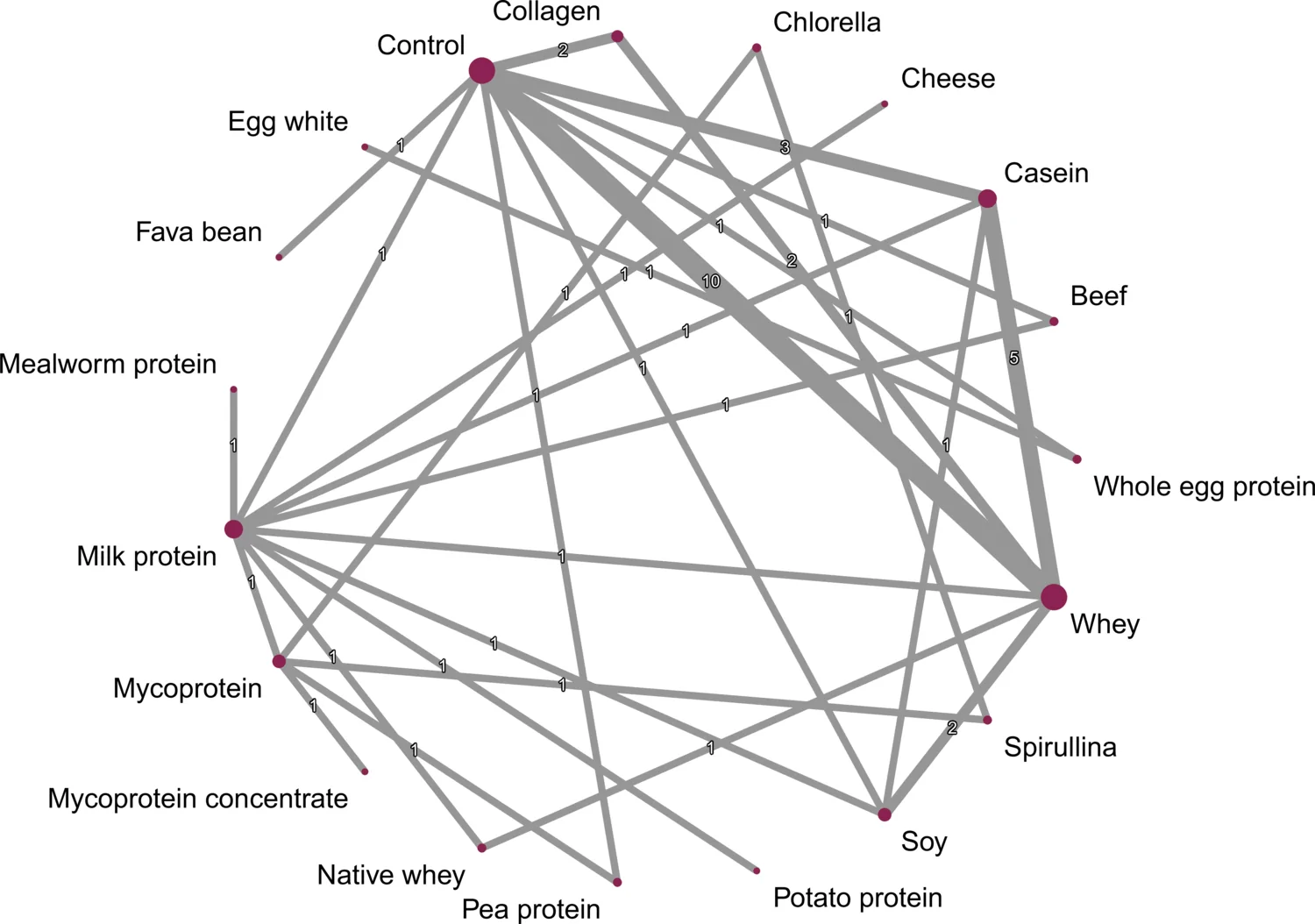
Network geometry plot.

#### 3.1.1. Standard NMA results

Standard NMA shows that the native whey (MD = 0.05, CI: 0.00; 0.10), pea protein (MD = 0.05, CI: −0.02; 0.11), and whole egg protein (MD = 0.04, CI: −0.01; 0.09) MPS ranked highest for estimated MPS effects but non-statistically significant (confidence intervals including 0 or below 0). Only whey protein demonstrated a statistically significant effect on the hourly MPS (MD = 0.02, CI: 0.01; 0.04) (Figure 3). A league table to demonstrate direct and network estimates has been created (Table S3). P-scores derived from the NMA model indicated the highest relative efficacy for native whey (0.81), followed by pea protein (0.77) and whole egg protein (0.72) (Table 2). Notably, native whey and pea protein were each represented by one and two study arms, respectively; estimates for these sources should be interpreted with particular caution.

**Figure 3.**
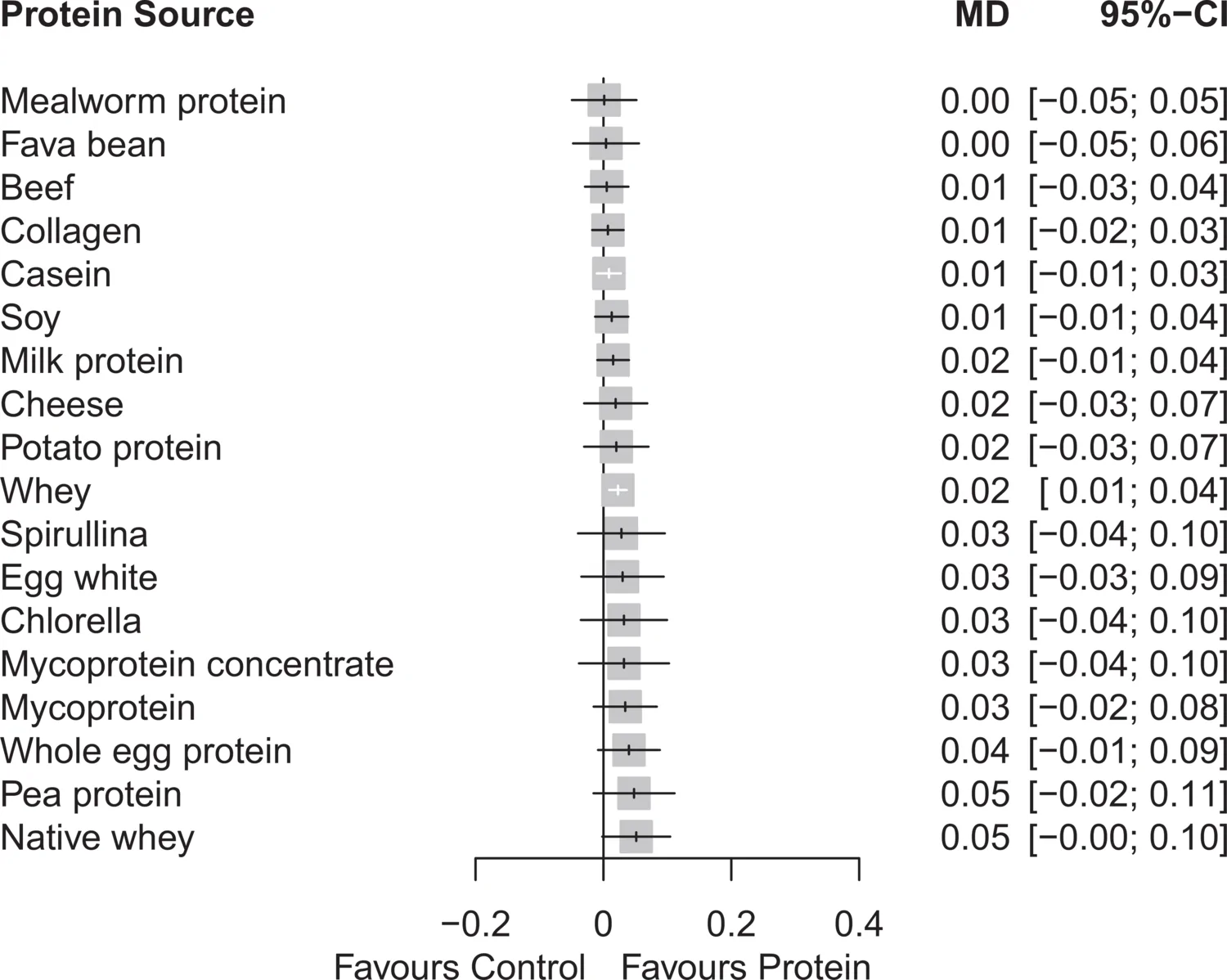
Forest plot illustrating MD and 95% CI of MPS rates of varying protein sources.

**Table 2.** Summary of findings from standard NMA demonstrating most likely effective protein sources to enhance muscle protein synthesis together with preceding resistance training.

Protein source	P-score (ranking)	Absolute effect estimates of MPS compared to placebo MD (95% CI)	Certainty of the evidence
Native whey	0.81	0.05 (0.00; 0.10)	⨁⨁⨁◯ Moderate^a^
Pea protein	0.77	0.05 (−0.02; 0.11)	⨁⨁⨁◯ Moderate^a^
Whole egg protein	0.72	0.04 (−0.01; 0.09)	⨁⨁⨁◯ Moderate^a^
Mycoprotein	0.66	0.03 (−0.02; 0.08)	⨁⨁⨁◯ Moderate^b^
**Whey**	**0.58**	**0.02 (0.01; 0.04)**	**⨁⨁◯◯ Low** ^a^ ^,^ ^c^

Note: Results are network meta-analysis effect estimates and 95% CIs for comparison against placebo/control intervention. P-scores provide a measure of the relative ranking of interventions, representing the extent to which an intervention is likely to be the most effective among all comparators (for example, a P-score of 0.81 indicates an 81% probability that the intervention is among the most effective options). Protein sources in bold are statistically significant. Mean differences (MD) represent hourly muscle protein synthesis rates (MPS [%//h]).

^a^ Downgrade due to risk of bias.

^b^ Imprecision.

^c^ Inconsistency.

The global test for inconsistency (i.e. design-by-treatment interaction random effects model) indicated inconsistency (Q = 35.70, *p*< 0.001). Local node-splitting analyses identified a source of inconsistency between direct and indirect evidence for the comparisons between casein vs control (*p* = 0.041) and casein vs soy (*p* = 0.013) (Table S2). While small differences between direct and indirect estimates were also observed in other comparisons, no additional statistically significant inconsistencies were found (Figure S4). As shown in Figure S6, the greatest contributions to inconsistency were observed in network loops involving casein and soy, which is consistent with the results of the local node-split analyses. Visual inspection of the comparison-adjusted funnel plot (Figure S5) indicated symmetry, suggesting that there was no evidence of small study effects and publication bias. Subgroup NMA analyses by age, sex, and type of protein synthesis were performed, but owing to sparse data, no formal conclusions can be drawn (Figures S7–S9). The sensitivity analysis demonstrated high heterogeneity when excluding high risk of bias and crossover trials (both I^2^ = 94%, *p*< 0.01).

#### 3.1.2. DR-NMA results

A forest plot with the DR-NMA results is shown in Figure S1. All effect estimates were based on predictions at the common dose of each protein source, defined as the median dose observed across studies. The results showed that native whey at its common dose of 20 g (MD = 0.06, CI: 0.02; 0.09) showed the highest predicted MPS rates at its common dose, followed by whole egg protein at 18 g (MD = 0.04, CI: 0.03; 0.06) and pea protein at 25 g (MD = 0.04, CI: 0.02; 0.06). A comparison between the standard NMA and DR-NMA results is shown in Figure S2, highlighting narrower confidence intervals in the DR-NMA, indicating greater precision. Protein sources with the lowest ability to stimulate MPS in both models were fava bean (NMA: MD = 0.00, CI: −0.05; 0.06. DR-NMA: MD = 0.00, CI: −0.03; 0.04) and mealworm protein (NMA: MD = 0.00, CI: −0.05; 0.05. DR-NMA: MD = 0.01, CI: −0.01; 0.03). Dose‒response curves for all protein sources are shown in Figure S3, providing a visual summary of the predicted effects across the dose range. The patterns varied across protein sources, with some showing an apparent increase in MPS with higher doses (e.g. native whey, whole egg protein, pea, and mycoprotein), while others exhibited flatter trends (especially mealworm and fava bean protein).

P-scores derived from the random-effects model indicated the highest relative efficacy for native whey (0.81), followed by pea protein (0.77), and whole egg protein (0.72), which aligns with the rankings observed under the fixed-effects model.

### 3.2. Risk of bias assessment

18% of the studies exhibited low, 72% moderate and 10% high risk of bias overall (Figures 4 and 5). Bias due to deviations from intended interventions (2nd domain), missing outcome data (3rd domain) and in measurement of outcome data (4th domain) was low in each study. Bias arising from the randomization process (1st domain) was considered high in one trial and moderate in 37 (of 51). The majority of studies failed to provide information on whether the allocation sequence was concealed, resulting in an classification of moderate risk of bias. Bias in the selection of the reported result (5th domain) was high in 4 and moderate in 24 studies. Approximately a third of the trials have not stated their pre-specified analysis plan preceding the availability of unblinded outcome data. In each of the four trials assessed as high risk, the registered protocols indicated that the results were likely selected from multiple eligible analyses of the data.

**Figure 4.**
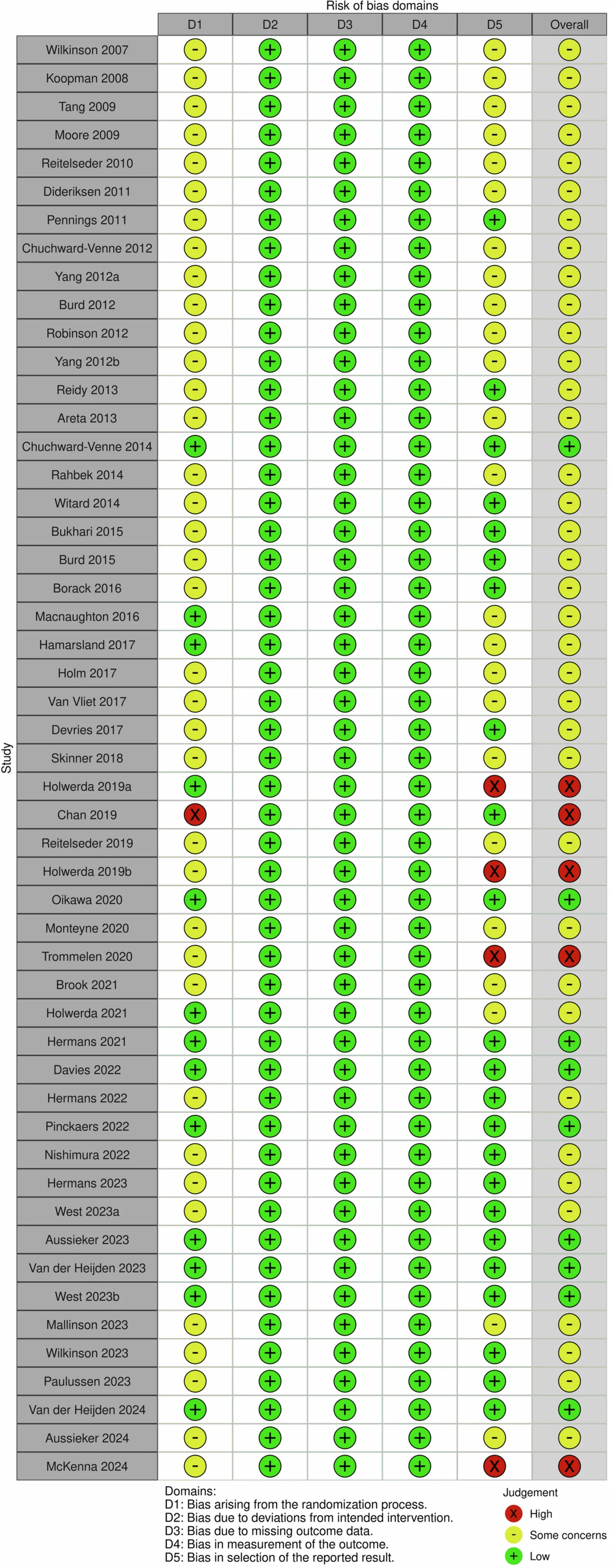
Detailed risk of bias assessment for each study.

**Figure 5.**
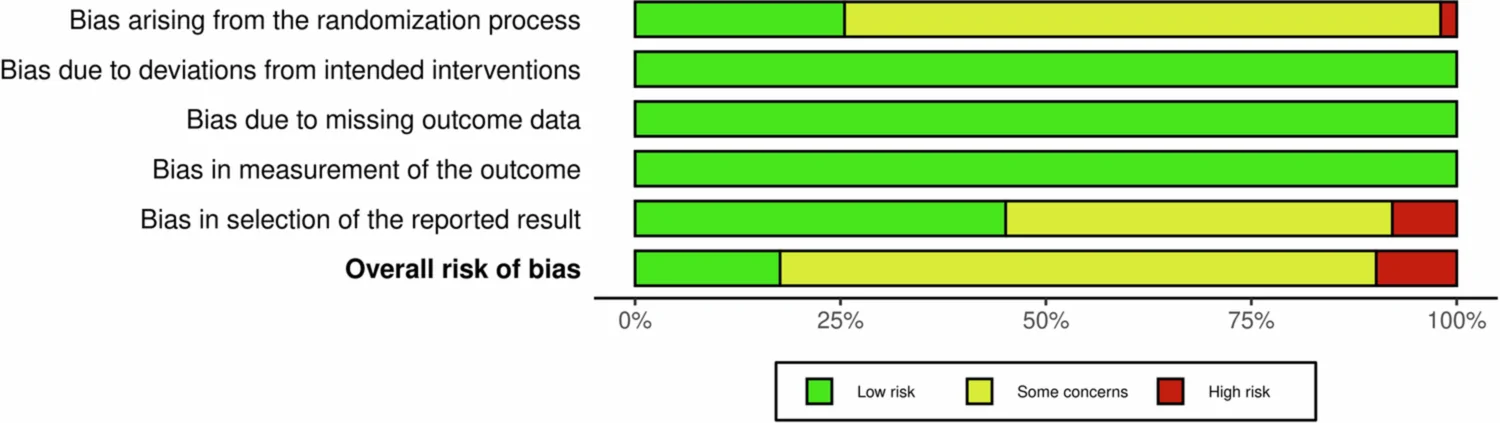
Overall risk of bias per domain.

## 4. Discussion

The present systematic review and network meta-analysis found native whey, pea protein, whole egg protein, mycoprotein and whey protein to rank highest estimated acute MPS rates per hour when combined with RT, with whey protein emerging as the only source demonstrating a statistically significant effect. These findings are consistent with the dose–response analysis, in which native whey (20 g), whole egg protein (18 g), pea protein (25 g), and mycoprotein (25 g) demonstrated showed the highest predicted MPS rates at their common doses (i.e. the median observed doses). These effects were statistically significant, and the agents were ranked in descending order of efficacy.

Contrary to our initial hypothesis, the findings suggest comparable efficacy between some plant- and animal-derived protein sources in stimulating MPS within a range of 18–25 g. It is important to note, however, no direct comparison of animal versus plant proteins as categories was conducted in this analysis; this observation is based on the relative rankings of individual protein sources within the network. This aligns particularly with the dose–response analysis, where both pea protein and mycoprotein (at 25 g) ranked among the top five statistically significant protein sources and demonstrated steadily increasing MPS rates with higher doses (Figure S3). This is consistent with recent meta-analyses that have not found significant differences in lean mass or strength gains [39] and MPS (at least in younger adults [40]) between plant- and animal-based proteins. Nonetheless, other conventional [41] and network [42] meta-analyses have identified milk protein as more effective than other protein types—particularly non-soy plant proteins such as rice, chia, oat, and potato—in promoting muscle mass and strength. In the present dose‒response NMA, pea protein and mycoprotein achieved comparable MPS rankings only at doses of 25 g, whereas native whey was effective at 18–20 g. This dose differential is consistent with the established lower digestibility and bioavailability of plant proteins, attributed to anti-nutritional factors such as proteinase inhibitors [43], fibrous matrices [44], hydrophobic character and limited structural flexibility [45], as well as reduced essential amino acid (EAA) and leucine content. Nevertheless, the present analysis does not formally compare doses between protein types, and no direct animal-versus-plant dose‒equivalence test was conducted; these observations are based on individual source rankings within the network and should be interpreted as hypothesis-generating.

The present analysis focused exclusively on hourly MPS rates measured within 2–7.5 h following a RT bout and subsequent protein ingestion. Studies assessing daily (“integrated”) MPS rates using doubly labeled water were excluded because of substantial heterogeneity in study designs and the limited number of available trials; however, long-term interventions aimed at enhancing MPS remain essential for promoting muscle mass accretion and its associated benefits. Two one-week trials involving healthy RT experienced young males resistant to exercising three to four times and supplementing animal-derived ~26 g of whey [46] and 30 g of collagen peptides [47] per day yet showed no significant increases in myofibrillar and connective tissue daily FSR, even though the comparator group ingested an isonitrogenous formulation of non-EAAs or non-caloric placebo. Similarly, a two-week and two ten-week trials involving regular resistance training combined with daily intake of 30 g peanut protein [48,49] and 50 g of potato protein [50] also failed to demonstrate increased daily MPS rates Compared to non-protein or non-food controls. It has been speculated that the absence of significant adaptations may be attributable to already high protein intake prior to and during the interventions—particularly relevant when studying highly trained individuals with elevated protein requirements [51]. Furthermore, the timing of MPS assessment—whether in close proximity to or long after the last exercise bout—may have influenced the integrated MPS response. Hence, daily MPS should not be interpreted as a “simple linear extension” of acute, hourly MPS rates, as it reflects a composite measure over time that includes periods of fasting, rest, and inactivity. Whether acute hourly MPS rates reliably predict long-term muscle hypertrophy remains debated [52–55], and the present findings should be interpreted accordingly.

Given the underrepresentation of women in the literature, a pooled analysis comparing MPS responses between sexes would be valuable. However, owing to the limited availability of sex-specific data, a quantitative analysis was not feasible. Nonetheless, visual inspection of the subgroup forest plot (Figure S8) suggested no substantial differences between males and females. This aligns with a recent meta-analysis reporting that females have a similar potential to induce muscle hypertrophy as males do [56]. A recent dose‒response RCT in women showed that post-exercise muscle mRNA expression related to protein turnover, substrate metabolism, remodeling, and MPS did not differ between whey protein drinks containing 0.6, 1.5, or 2 g of leucine, suggesting that current protein/leucine recommendations may already suffice to optimally stimulate MPS, at least in young females [57]. Further evidence suggests that middle-aged women (~45 years) may exhibit a greater acute myofibrillar protein synthetic response to the ingestion of 25 g of whey protein compared to their male counterparts do [58]. In obese older adults (65–80 years), a three-month exercise training intervention resulted in similar postprandial MPS rates in men and women. Notably, the basal MPS in women was already elevated before and after the intervention, suggesting a blunted meal-induced MPS response [59]. Owing to the limited sample sizes, future studies with larger cohorts are warranted to confirm these results.

The present results of the network meta-analysis should be interpreted with caution due to some arising limitations. Subgroup analyses were performed, but the available data within subgroups were too limited to allow for reliable comparisons, underscoring the substantial need for adequately powered studies stratified by age and sex. Additionally, certain indirect comparisons were not estimable due to incomplete network geometry. While some protein sources were frequently studied, most trials have examined a wide range of diverse protein interventions, thereby limiting their statistical power. The pronounced heterogeneity likely arises from differences in study design, participant characteristics (e.g. training status), exercise protocols, protein sources and doses, and the timing of muscle protein synthesis assessment. Furthermore, outcomes related to the most effective proteins were affected by concerns regarding the risk of bias, imprecision, and inconsistency. Furthermore, the global inconsistency test indicated significant inconsistency in the network (Q = 35.70, *p* < 0.001), which was driven primarily by comparisons involving casein. This means that direct and indirect network estimates may not be fully coherent. The high degree of heterogeneity (I^2^ = 92.7%) further reduces confidence in the pooled estimates. Several protein sources with high P-score rankings (native whey: one study arm; pea protein: two study arms) were represented by very limited evidence, underscoring that treatment rankings should not be interpreted independently of the quantity and quality of underlying evidence. Furthermore, the majority of included studies were conducted in young, healthy adults, limiting the generalizability of the present findings to older populations; older adults are known to exhibit reduced anabolic sensitivity to dietary protein and resistance exercise (“anabolic resistance”) [60], which may substantially alter protein supplement efficacy rankings in this population.

In conclusion, both animal- (specifically native whey, whey, and whole egg protein) and plant-derived (in particular pea and mycoprotein) protein sources, when combined with resistance training, are ranked similarly for estimated acute MPS stimulation. However, some plant-derived sources (notably pea and mycoprotein) showed comparable rankings only at doses of 25 g, which exceeded common doses for animal proteins (18–20 g), suggesting that higher absolute doses may be required; direct comparative evidence is needed to confirm this. These effects may apply across sexes and age groups, as similar patterns have been qualitatively observed in both. However, further trials are warranted to statistically confirm these preliminary findings. Enhancing the MPS both acutely and chronically is essential for promoting and preserving skeletal muscle mass [61] across the lifespan, thereby supporting metabolic health and longevity [62].

## Supplementary Material

Supplementary Material Supplementary_files.zip

## Data sharing statement

The datasets generated during the review are available from the corresponding author upon reasonable request. Table S1 contains relevant data.
