# Supplementary material for: Impact of different protein sources on acute muscle protein synthesis following resistance training: a systematic review with standard and dose–response network meta-analyses
Source: J Int Soc Sports Nutr. 2026 Oct 12;23(1):2740224. doi: 10.1080/15502783.2026.2740224 (PMC13660545; doi:10.1080/15502783.2026.2740224)
Supplement: Supplementary file 1 [file RSSN_A_2740224_SM5642.zip › Supplementary captions.docx]

Figure S1: Forest plot demonstrating the DR-NMA estimates of MD and 95% CI. Predictions were based on the common dose of each protein source, defined as the median dose observed across the included studies.

Figure S2: Forest plot comparing "standard" and dose-response network meta-analysis. Doses in parentheses represent median observed doses (common doses) used for prediction in the DR-NMA model.

Figure S3: Dose-response plots showing MPS rates for each protein source (dose). Green dots indicate direct observed comparison with the reference whereas red dots illustrate indirect observed comparison with the reference. Blue lines represent mean difference with the 95% confidence interval in grey.

Figure S4: Forest plot with node-splitting approach to identify direct, indirect and network estimate.

Figure S5: Comparison-adjusted funnel plot for visual inspection of potential publication bias.

Figure S6: Net-heat plot.

Figure S7: Subgroup analysis forest plot regarding age.

Figure S8: Subgroup analysis forest plot regarding sex.

Figure S9: Subgroup analysis forest plot regarding measured type of protein synthesis.
