# Supplementary material for: Impact of different protein sources on acute muscle protein synthesis following resistance training: a systematic review with standard and dose–response network meta-analyses
Source: J Int Soc Sports Nutr. 2026 Oct 12;23(1):2740224. doi: 10.1080/15502783.2026.2740224 (PMC13660545; doi:10.1080/15502783.2026.2740224)
Supplement: Supplementary file 1 [file RSSN_A_2740224_SM5642.zip › supplementary file1.docx]

**Supplementary File 1**

Impact of different protein sources on acute muscle protein synthesis following resistance training: a systematic review with standard and dose–response network meta-analyses

Kevin Bischof ^2,3*^, Anna Maria Moitzi ^1,3^, Maria Petropoulou ^4^, Brendon Stubbs ^2,5^, Daniel König ^1,2,3^

^1^ Faculty of Life Sciences, Department for Nutrition, Section for Nutrition, Exercise and Health, University of Vienna, Vienna, Austria

^2^ Centre for Sports Science and University Sports, Department of Sports Science, Section for Nutrition, Exercise and Health, University of Vienna, Vienna, Austria

^3^ Vienna Doctoral School of Pharmaceutical, Nutritional and Sport Sciences, University of Vienna, Vienna, Austria

^4^ Institute of Medical Biometry and Statistics, Faculty of Medicine and Medical Center – University of Freiburg, Freiburg, Germany

^5^ Department of Psychological Medicine, Institute of Psychiatry, Psychology and Neuroscience (IoPPN), King's College London, London, UK

*** Correspondence:**
Kevin Bischof, <https://orcid.org/0009-0008-6416-6524>	[kevin.bischof@univie.ac.at](mailto:kevin.bischof@univie.ac.at)

**Model Evaluation Report**

We applied several models, including the Network Meta-Analysis (NMA), as well as various dose-response NMA models, such as:

- Linear, Exponential, and Quadratic Polynomial models
- Fractional Polynomial (FP1) transformations with power pfrom the set S = {-2, -1, -0.5, 0, 0.5, 1, 2, 3}
- Restricted Cubic Splines (RCS) with knots at k = c(0.1, 0.5, 0.9) (RCS) and k = c(0.25, 0.5, 1)) (RCS2)

Model fit was evaluated based on the Q/df ratio, which quantifies how well each model explains heterogeneity while avoiding unnecessary complexity.

**Summary of Model Performance**

- FP1 (p = 0.5) and FP1 (p = 0) had the lowest Q/df ratio (9.79 and 9.83, respectively), indicating that they provide the best fit and explain heterogeneity most efficiently.
- The Quadratic Polynomial model ranked third with Q/df = 10.63, suggesting a good balance between flexibility and model complexity.
- Linear, RCS, and FP1 (p = 1) showed similar results with Q/df ~10.87, meaning they offer comparable performance in explaining heterogeneity.
- The Exponential and the RCS2 model had a slightly higher Q/df ~ 11.27, making it a less favorable option.
- The classic NMA model (Q/df = 13.65) exhibited higher heterogeneity, indicating a poorer model fit compared to the above dose-response models.
- FP1 (p = 2) and FP1 (p = 3) showed the highest Q/df values (14.62 and 21.23, respectively), suggesting that they do not fit the data well and introduce excessive complexity.
- FP1 (p = -0.5) ranked just above FP1 (p = -1 and -2), with Q/df = 15.36, suggesting poor performance compared to other fractional polynomial models.
- FP1 (p = -1) had a very high Q/df = 19.20, indicating substantial heterogeneity that remains unexplained.
- FP1 (p = -2) had the worst fit, with Q/df = 21.63 and extremely high heterogeneity making it the least suitable model.

Based on the model comparison analysis, FP1 (p = 0.5) demonstrated the best fit, with the lowest Q/df ratio (9.79).

**Results (additional information)**

As shown in Figure 1, of the remaining 52 articles, one full-text could not be obtained despite written correspondence with the authors, who did not respond to the request.[1] An additional eleven full-text articles were excluded due to gauging a wrong outcome parameter (n=7), absence of any form of resistance exercise preceding muscle biopsy and protein intake (n=3), provision a whole meal (n=1), measuring MPS (baseline to post workout) several hours post-exercise following overnight sleep (n=1). Of the 34 retrieved full-text articles derived from non-search string individual search (Google Scholar & references), 22 papers were not considered for final inclusion for the following reasons: missing exercise intervention (n=11), wrong outcome parameter (n=3), representing a non-randomized study of interventions (n=1), collection of 24-hour MPS (n=1), being a review (n=1), missing protein source (n=4), provision of a whole meal plus additional protein supplement (n=1).

Table S1: Overview of included studies.

| Authors | Year | Cross-over (CO)? | N | Age | Gender (m/f) | Training status | Exercise intervention | Protein source + dosage | Type of MPS | Duration of MPS | Amino acid tracer | Mean ± SD |
| --- | --- | --- | --- | --- | --- | --- | --- | --- | --- | --- | --- | --- |
| *Reidy et al [2]* | *2013* |  | *19* | *18-30* | *17/2* | *recreationally active* | *leg extension 8x10 (55-70% 1RM)* | *Whey ~18g (n=9)* | *mixed/h* | *4h* | *L-[ring-13C6] phenylalanine* | *0.077 ± 0.018* |
| Davies et al [3] | 2022 |  | 16 | 18-35 | 8/8 | *recreationally active* | knee extension (KE) 6x10 | Fava Bean 0,33 g/kg (*n=*8), CON: no EAAs (*n=*8) | myo/h | 3h | deuterium labelling of myofibrillar-bound alanine | fava:0.091 ± 0.02 con:0.087 ± 0.039 |
| *Heijden et al [4]* | *2024* | *CO* | *10* | *26* | *8/2* | *RE trained* | *3 leg exercises each 4x10* | *Whey 32g* | *myo/h* | *4h (0-2, 2-4, 0-4)* | *L-[ring-2H5] phenylalanine* | *whey 0-2h: 0.085 ± 0.037 whey 2-4h: 0.085 ± 0.036 whey 0-4h: 0.084 ± 0.02* |
| *Devries et al [5]* | *2018* |  | *22* | *65-75* | *0/22* | *Low to moderate PA* | *RE 4 sets (50-60 1RM)* | *whey protein isolate 25g (n=11)* | *myo/h* | *4h* | *13C6-ring phenylalanine* | *0.051 ± 0.007* |
| *Holwerda et al [6]* | *2019* |  | *24* | *~69* | *24/0* | *Moderatetly active* | *60 min whole body RE (50-80% 1RM)* | *Milk Protein Concentrate 15g (n=12)* | *myo/h* | *6h* | *L-[ring-2H5]phenylalanine (taken for analysis), L-[1-13C] leucine* | *Phenylal.: 0.0495 ± 0.0021 Leucine: 0.0598 ± 0.003* |
| Hamarsland et al [7] | 2017 | Partial CO | 22 | ~25 | 13/9 | strength trained | 4x8 leg press and KE | Milk 20g (n=12), Native whey 20g (n=10), Whey (WPC-80) 20g (n=10) (whey and WPC-80 crossover), supplements given 0 und 120min after RE | mixed/h | 4h (1-3, 3-5,1-5) | [2H5] phenylalanine | milk 1-5h: 0.064 ± 0.033 wpc 1-5h: 0.102 ± 0.052 native 1-5h: 0.112 ± 0.049 milk 1-3h: 0.081 ± 0.047 wpc 1-3h: 0.12 ± 0.046 native 1-3h: 0.11 ± 0.066 milk 3-5h: 0.067 ± 0.042 wpc 3-5h: 0.071 ± 0.064 native 3-5h: 0.111 ± 0.064 |
| *Churchward-Venne et al [8]* | *2012* |  | *24* | *~22* | *24/0* | *recreationally active* | *KE and leg-press 4x10-12* | *Whey isolate 25g (n=8)* | *myo/h* | *2h (1-3, 3-5)* | *[ring-13C6] phenylalanine* | *1-3h :0.064 ± 0.007 3-5h :0.088 ± 0.01* |
| Churchward-Venne et al [9] | 2014 |  | 40 | 18-35 | 40/0 | *recreationally active* | KE 8x10-12 | whey protein 25g (n=8), whey 6g (n=8) | myo/h | 4.5h | [ring-13C6] phenylalanine | whey 6g: 0.0469 ± 0.017 whey 25g: 0.06 ± 0.011 |
| Wilkinson et al [10] | 2007 | CO | 8 | ~22 | 8/0 | RE trained | leg press, hamstring curl and KE, 4x 80% 1RM | isolated soy protein 18g (n=4), skim milk powder 18g (n=4) | mixed/h | 3h | L-[ring2 H5] phenylalanine | milk: 0,1 ± 0.039 soy: 0,066 ± 0.039 |
| Oikawa et al [11] | 2020 |  | 22 | 60-80 | 0/22 | Low PA | 4 x 8-10 RE legs | Whey 30g (n=11), collagen 30g (n=11) | myo/h | 4h | L-[ring−13C6] phenylalanine | whey: 0.051 ± 0.011 col: 0.035 ± 0.011 |
| *Brook et al [12]* | *2021* | *CO* | *8* | *~71* | *8/0* | *no former exercise regimes* | *KE 6x8 (75% 1RM)* | *milk protein 15g (n=4)* | *myo/h* | *4h* | *15N-Phenylalanine* | *0.093 ± 0.013* |
| Yang et al [13] | 2012 |  | 50 | ~71 | 50/0 | light to-moderately active | KE 3 sets 10RM | water, soy 20g, soy 40g, whey 20g, whey 40g, each n=10 | myo/h | 4h | L-[ring-13C6] phenylalanine | water: 0.031 ± 0.007 soy 20g: 0.041 ± 0.01 whey 20g: 0.055 ± 0.009 soy 40g: 0.056 ± 0.009 whey 40g: 0.082 ± 0.026 |
| Burd et al [14] | 2012 |  | 14 | ~72 | 14/0 | light to-moderately active | KE 3x10RM | micellar casein 20g (n=7), whey protein isolate 20g (n=7) | myo/h | 4h | L-[ring-13C6] phenylalanine | micellar: 0.035 ± 0.002 whey: 0.055 ± 0.005 |
| *Bukhari et al [15]* | *2015* |  | *16* | *~66* | *0/16* | *No formal strenuous exercise regimes* | *KE 6x8 (75% 1-RM)* | *whey protein 20g (n=8)* | *myo/h* | *4h* | *L-[ring-13C6] phenylalanine* | *0.093 ± 0.007* |
| Tang et al [16] | 2009 |  | 18 | ~23 | 18/0 | RE trained | 4x leg press and KE 10-12RM | whey 21.4g (n=6), casein 21.9g (n=6), soy 22.2g (n=6) | mixed/h | 3h | L-[ring-13C6] phenylalanine | whey: 0.151 ± 0.017 casein: 0.069 ± 0.006 soy: 0.115 ± 0.01 |
| Reitelseder et al [17] | 2011 |  | 17 | ~27 | 17/0 | moderately active | 10x8 leg extension | whey ~20g (n=9), casein ~20g (n=9), water (n=8) | myo/h | 6h | L-[1-13C] leucine | water: 0.073 ± 0.025 whey: 0.097 ± 0.021 casein: 0.102 ± 0.024 |
| Dideriksen et al [18] | 2011 |  | 24 | 61-80 | 15/9 | moderately active | 5x8 KE and leg press | Casein (~23g), whey (~25g), control (0g), each n=6 | myo/h col/h | 6h | L-[1-13C] leucine | myo_water: 0.07 ± 0.006 myo_casein:0.09 ± 0.003 myo_whey: 0.09 ± 0.005 col_water: 0.04 ± 0.002 col_casein:0.06 ± 0.006 col_whey: 0.06 ± 0.006 |
| *Areta et al [19]* | *2013* |  | *24* | *~25* | *24/0* | *RE trained* | *4x10 leg extensions* | *whey protein isolate 40g (n=8)* | *myo/h* | *1-4h* | *L-[ring-13C6] phenylalanine* | *0.068 ± 0.012* |
| *Robinson et al [20]* | *2012* |  | *35* | *~59* | *35/0* | *healthy* | *3x8-10 KE* | *Control, 57g (=12g), 113g (=24g), 170g (=36g), each n=7* | *myo/h* | *0-4h* | *L-[ring-13C6] phenylalanine* | *Control: 0.031 ± 0.0098 beef 57g: 0.035 ± 0.0097 beef 113g: 0.042 ± 0.007 beef 170g: 0.062 ± 0.034* |
| *Hermans et al [21]* | *2023* |  | *29* | *18-34, 65-85* | *29/0* | *recreationally active* | *4x8-10 KE and leg press* | *291 g (=30g protein) quark* | *mixed/h* | *0-4h* | *L-[ring-13-C6] phenylalanine* | *young: 0.071 ± 0.023 elderly: 0.078 ± 0.019* |
| West et al [22] | 2023 |  | 33 | ~21 | 24/9 | RE trained | whole body, 4x each exercise at 10RM | mycoprotein 48g (=25g), pea protein 32g (=25g), each n=11 | myo/h | 0-4h | L-[ring-2H5] phenylalanine | myc: 0.076 ± 0.004 pea: 0.087 ± 0.01 |
| Aussieker et al [23] | 2023 |  | 45 | ~25 | 30/15 | *Recreationally active* | Squats 6x8-15 | whey 30g, col. hydrolysate 30g, non-caloric placebo 30g, each n=15 | myo/h col/h | 0-5h | L-[ring-13C6] phenylalanine | myo_pla: 0.032 ± 0.007 myo_coll: 0.036 ± 0.01 myo_whey: 0.040 ± 0.01 col_pla: 0.058 ± 0.018 col_col: 0.068 ± 0.017 col_whey: 0.072 ± 0.019 |
| Hermans et al [24] | 2022 |  | 20 | 18-35 | 20/0 | healthy | 6x ~8-10 KE unilateral | cheese 103g (=30g), milk protein concentrate 37g (=30g), each n=10 | mixed/h | 0-4h | L-[ring-13C6] phenylalanine | cheese: 0.067 ± 0.013 milk conc.: 0.063 ± 0.009 |
| Heijden et al [25] | 2023 |  | 36 | ~22 | 18/18 | *Recreationally active* | 4x8-12 KE & leg press | protein content (25 g), 46.7, 40.4, and 42.0 g of MYCO, SPIRUL, and CHLOR powders, each n=12 | myo/h | 0-4h | L-[ring-2H5] phenylalanine | myco.: 0.092 ± 0.024 spirul.: 0.086 ± 0.028  chlor.: 0.090 ± 0.024 |
| Chan et al [26] | 2019 |  | 30 | 18-35 | 30/0 | not RT experienced | 3x10 leg press & extensions | Milk Protein Concentrate 25g, calcium caseinate 25g, each n=10 | myo/h | 0-4h | l-[ring-13C6] phenylalanine | mpc: 0.0562 ± 0.024 casein: 0.0598 ± 0.025 |
| Reitelseder et al [27] | 2019 |  | 27 | >60 | 27/0 | moderately active | 10x8 leg extension | whey hydrolysate (n=10), caseinate (n=9), carbs (n=8) each 0,45g/kg | myo/h | 0-3h | L-[15N] phenylalanine | whey: 0.041 ± 0.012 casein: 0.043 ± 0.012 carbs: 0.034 ± 0.011 |
| *Holwerda et al [28]* | *2021* |  | *36* | *~70* | *36/0* | *healthy* | *6x 55-75% 1RM leg extensions & press* | *casein 40g (n=9)* | *col/h* | *0-7.5h* | *L-[ring-2H5] phenylalanine* | *0.095 ± 0.022* |
| Hermans et al [29] | 2021 |  | 24 | ~23 | 24/0 | healthy | 5x 50-80% 1RM leg extensions & press | milk protein 30g (n=12), mealworm–derived protein 30g (n=11) | mixed/h | 0-5h | l-[ring-2H5] phenylalanine | worm: 0.059 ± 0.015 milk: 0.073 ± 0.020 |
| Monteyne et al [30] | 2020 |  | 20 | ~22 | 20/0 | *RE trained* | 5x 30 leg flexions & extensions | milk protein 26.2 g (n=10), mycoprotein 31.5 g (n=9) | mixed/h | 0-4h | L-[ring-2H5] phenylalanine | milk: 0.056 ± 0.016 myco: 0.072 ± 0.015 |
| *Trommelen et al [31]* | *2020* |  | *36* | *~23* | *36/0* | *recr. active* | *6x10 leg press & extensions* | *casein 30g (n=12)* | *col/h* | *0-7.5h* | *L-[ring-2H5] phenylalanine* | *0.080 ± 0.019* |
| Holm et al [32] | 2017 |  | 20 | ~24 | 20/0 | *recreationally active* | 6x10 KE | 0.3g whey + 0.3g CHO/kg LBM, 0.6g CHO/kg LBM, each n=10 | col/h | 1-5h | Ring-13C6 phenylalanine | whey: 0.119 ± 0.028 carbs: 0.11 ± 0.032 |
| *Borack et al [33]* | *2016* |  | *20* | *55-75* | *20/0* | *recreationally active* | *8x10 (to 70% 1RM) KE* | *whey protein isolate 30.4g (n=10)* | *mixed/h* | *0-4h* | *L-[ring-13C6] phenylalanine* | *0.081 ± 0.025* |
| Burd et al [34] | 2015 | CO | 12 | ~22 | 12/0 | recreationally active | 4x8-10 KE and leg press | 158g (=30g) minced beef patty, skim milk powder 30g, each n=12 | myo/h | 0-5h | L-[1-13C] phenylalanine | beef: 0.057 ± 0.021 milk: 0.071 ± 0.017 |
| van Vliet et al [35] | 2017 | CO | 10 | ~21 | 10/0 | *RE trained* | 4x10 leg press & extensions | egg white, whole eggs, each 18g & n=10 | myo/h | 0-5h | L-[ring-2H5] phenylalanine | whole egg: 0.034 ± 0.013 egg white: 0.024 ± 0.006 |
| Moore et al [36] | 2009 | CO | 6 | ~22 | 6/0 | RE trained | 4x8-10 leg press, KE and leg curl | whole egg protein 0g, 5g, 10g, 20g,40g each n=6 | mixed/h | 0-4h | [1-13C] leucine | 0g: 0.053 ± 0.017 5g : 0.0749 ± 0.021 10g: 0.0859 ± 0.026 20g: 0.107 ± 0.027 40g: 0.115 ± 0.027 |
| Yang et al [37] | 2012 |  | 37 | ~71 | 37/0 | light-to-moderately active | 3x 10RM KE | whey 0g (n=10),10g (n=7), 20g (n=9), 40g (n=10) | myo/h | 0-4h | L-[ring-13C6] phenylalanine | 0g: 0.043 ± 0.013 10g: 0.0491 ± 0.004 20g: 0.062 ± 0.009 40g: 0.082 ± 0.032 |
| Witard et al [38] | 2014 |  | 48 | ~21 | 48/0 | RT experienced | 8x10 leg press & extensions | whey 0g, 10g, 20g, 40g, each n=12 | myo/h | 0-4h | L-[ring-13C6] phenylalanine | 0g: 0.041 ± 0.052 10g: 0.0597 ± 0.011 20g: 0.069 ± 0.01 40g:0.076 ± 0.01 |
| *Macnaughton et al [39]* | *2016* | *CO* | *30* | *~22* | *30/0* | *RE trained* | *whole body workout, 4 sets each exercise (~75% 1RM)* | *whey isolate 20g, 40g each n=30* | *myo/h* | *0-5h* | *L- [ring-13C6] phenylalanine* | *20g: 0.049 ± 0.003 40g: 0.058 ± 0.003* |
| Holwerda et al [40] | 2019 |  | 48 | 55-80 | 48/0 | no structured RT program | whole body workout, sets varied (2-5), 50-80% 1RM | milk protein concentrate 0g, 15g, 30g, 45g, each n=12 | myo/h | 0-6h | L-[1-13C] leucine | 0g: 0.074 ± 0.017 15g: 0.084 ± 0.017 30g: 0.095 ± 0.021 45g: 0.097 ± 0.021 |
| Pinckaers et al [41] | 2022 |  | 24 | ~24 | 24/0 | recreationally active | KE 4x80% 1RM | potato protein concentrate 30g, milk protein concentrate 30g, each n=12 | mixed/h | 0-5h | L-[ring-13C6] phenylalanine | potato: 0.069 ± 0.019 milk: 0.064 ± 0.015 |
| West et al [42] | 2023 |  | 24 | ~21 | 24/0 | *RE trained* | 4x70% 1RM leg press & extensions | mycoprotein 70g (=31.4g), protein conc. from mycoprotein 38.2 (=28g), each n=12 | myo/h | 0-4h | L-[ring-2H5] phenylalanine | myco: 0.082 ± 0.035 pcmyco: 0.080 ± 0.035 |
| *Mallinson et al [43]* | *2023* |  | *24* | *~26* | *0/24* | *RE trained* | *whole body RT 3x8 (75% 1RM) each exercise* | *whey isolate 15, 30, 60g each n=8* | *myo/h* | *0-4h* | *deuterium labelling of myofibrillar-bound alanine* | *15g: 0.09 ± 0.051 30g: 0.140 ± 0.059 60g: 0.109 ± 0.031* |
| Wilkinson [44] | 2023 |  | 19 | ~22 | 8/11 | recreationally active | 10x30 KE | pea protein isolate 25g (n=10), placebo (n=9) | myo/h | 0-3h | [2H] alanine | placebo: 0.151 ± 0.213 pea: 0.288 ± 0.31 |
| *Paulussen et al [45]* | *2023* | *CO* | *10* | *~24* | *5/5* | Recr. active | *4x10 leg press & extensions* | *salmon 99g (=20g) (n=5)* | *myo/h* | *0-5h* | *L-[ring-2H5] phenylalanine* | *0.08 ± 0.024* |
| Aussieker et al (abstract only) [46] | 2024 |  | 45 | ~23 | 21/24 | Not available | RE | collagen 30g (n=15), placebo (n=15) | myo/h col/h | 0-6h | L-[ring-13C6] phenylalanine | pla_myo: 0.049 ± 0.01 col_myo: 0.048 ± 0.011 pla_col: 0.098 ± 0.023 col_col: 0.092 ± 0.028 |
| McKenna et al [47] | 2024 |  | 16 | 50-79 | 0/16 | healthy | 3x12 (65% 1RM) leg extensions | 0.29g/kg whey (n=8), water (n=8) | myo/h | 0-4h | L-[ring-13C6] phenylalanine | water: 0.1 ± 0.048 whey: 0.124 ± 0.114 |
| *Pennings et al [48]* | *2011* |  | *48* | *~21, 74* | *48/0* | *no former exercise regimes* | *6x10 (40-75% 1RM) leg press & extensions* | *20g casein* | *mixed/h* | *0-6h* | *L-[1-13C] phenylalanine* | *young: 0.076 ± 0.015 elderly: 0.074 ± 0.014* |
| *Nishimura et al [49]* | *2022* | *CO* | *10* | *~22* | *10/0* | *moderately RE trained* | *Leg press, KE, shoulder press, pull downs, 3x8-10* | *meat protein hydrolysate 0.6g/FFM (= ~36g)* | *myo/h* | *0.5-3h* | *L-[15N] phenylalanine* | *0.086 ± 0.043* |
| *Koopman et al [50]* | *2008* | *CO* | *8* | *~73* | *8/0* | *no former exercise regimes* | *6x10 leg press & extensions* | *Cho + whey protein hydrolysate) 0.16 g/kg/h = ~69g over 6h* | *mixed/h* | *0-6h* | *L-[ring-13C6] phenylalanine* | *0.082 ± 0.017* |
| Rahbek et al [51] | 2014 |  | 24 | 24 | 24/0 | recreationally active | 6x10 eccentric KE for one leg and 6x10 concentric for the other leg | 0.3g whey + 0.3g CHO/kg LBM, 0.6g CHO/kg LBM, each n=12 | myo/h | 1-3h | L-[ring-13C6] phenylalanine | control: 0.0975 ± 0.027 whey: 0.105 ± 0.035 |

Italics = Study of which single arms were extracted, KE = Knee extension, RM = Repetition maximum, RE = Resistance exercise, EAA = Essential amino acids, LBM = Lean body mass, CHO = Carbohydrates, CO = Crossover trial.

Table S2: Local node-splitting analyses.

| Comparison | TE | Lower | Upper | p |
| --- | --- | --- | --- | --- |
| Beef vs Control | 0.0095 | -0.0591 | 0.0782 | 0.7855 |
| Casein vs Control | 0.0393 | 0.0015 | 0.0770 | **0.0415** |
| Casein vs Milk protein | 0.0149 | -0.0419 | 0.0717 | 0.6075 |
| Casein vs Soy | -0.0701 | -0.1254 | -0.0147 | **0.0131** |
| Casein vs Whey | -0.0186 | -0.0633 | 0.0262 | 0.4159 |
| Collagen vs Control | -0.0110 | -0.0625 | 0.0405 | 0.6763 |
| Collagen vs Whey | 0.0183 | -0.0335 | 0.0702 | 0.4882 |
| Milk protein vs Control | 0.0024 | -0.0509 | 0.0557 | 0.9285 |
| Pea protein vs Control | 0.0958 | -0.1539 | 0.3455 | 0.4520 |
| Soy vs Control | 0.0087 | -0.0450 | 0.0623 | 0.7519 |
| Whey vs Control | -0.0336 | -0.0728 | 0.0056 | 0.0927 |
| Milk protein vs Mycoprotein | 0.0958 | -0.1539 | 0.3455 | 0.4520 |
| Milk protein vs Native whey | -0.0946 | -0.2495 | 0.0603 | 0.2312 |
| Milk protein vs Soy | 0.0452 | -0.0222 | 0.1127 | 0.1888 |
| Milk protein vs Whey | -0.0383 | -0.1010 | 0.0244 | 0.2312 |
| Mycoprotein vs Pea protein | 0.0958 | -0.1539 | 0.3455 | 0.4520 |
| Native whey vs Whey | -0.0742 | -0.1958 | 0.0473 | 0.2312 |
| Soy vs Whey | -0.0360 | -0.0895 | 0.0175 | 0.1867 |

Inconsistency between direct and indirect evidence for casein vs control (p = 0.041) and casein vs soy (p = 0.013) is apparent (in bolt). TE=treatment effect, seTE= Standard Error of the TE, lower and upper limit of the confidence interval. Other comparisons were not applicable.

Table S3: League table demonstrating direct (upper-right) and network (lower-left) estimates.

|  | Beef | Casein | Cheese | Chlorella | Collagen | Control | Egg white | Fava bean | Mealworm protein | Milk protein | Mycoprotein | Mycoprotein concentrate | Native whey | Pea protein | Potato protein | Soy | Spirullina | Whey | Whole egg protein |
| --- | --- | --- | --- | --- | --- | --- | --- | --- | --- | --- | --- | --- | --- | --- | --- | --- | --- | --- | --- |
| Beef | Beef | . | . | . | . | 0.01 [-0.04; 0.05] | . | . | . | -0.01 [-0.06; 0.03] | . | . | . | . | . | . | . | . | . |
| Casein | -0.00 [-0.04;  0.03] | Casein | . | . | . | 0.03 [ 0.00; 0.05] | . | . | . | 0.00 [-0.04; 0.05] | . | . | . | . | . | -0.05 [-0.09; 0.00] | . | -0.02 [-0.04; 0.00] | . |
| Cheese | -0.01 [-0.07;  0.04] | -0.01 [-0.06;  0.04] | Cheese | . | . | . | . | . | . | 0.00 [-0.04; 0.05] | . | . | . | . | . | . | . | . | . |
| Chlorella | -0.03 [-0.10;  0.04] | -0.02 [-0.09;  0.04] | -0.01 [-0.09;  0.06] | Chlorella | . | . | . | . | . | . | -0.00 [-0.05; 0.04] | . | . | . | . | . | 0.00 [-0.04; 0.05] | . | . |
| Collagen | -0.00 [-0.04;  0.04] | 0.00 [-0.03;  0.03] | 0.01 [-0.04;  0.07] | 0.03 [-0.05;  0.10] | Collagen | 0.00 [-0.03; 0.03] | . | . | . | . | . | . | . | . | . | . | . | -0.01 [-0.04; 0.02] | . |
| Control | 0.01 [-0.03;  0.04] | 0.01 [-0.01;  0.03] | 0.02 [-0.03;  0.07] | 0.03 [-0.04;  0.10] | 0.01 [-0.02;  0.03] | Control | . | -0.00 [-0.06; 0.05] | . | -0.02 [-0.06; 0.03] | . | . | . | -0.14 [-0.38; 0.10] | . | -0.02 [-0.06; 0.02] | . | -0.02 [-0.03; 0.00] | -0.04 [-0.09; 0.01] |
| Egg white | -0.02 [-0.10;  0.05] | -0.02 [-0.09;  0.05] | -0.01 [-0.09;  0.07] | 0.00 [-0.09;  0.10] | -0.02 [-0.09;  0.05] | -0.03 [-0.09;  0.03] | Egg white | . | . | . | . | . | . | . | . | . | . | . | -0.01 [-0.05; 0.03] |
| Fava bean | 0.00 [-0.06;  0.06] | 0.00 [-0.05;  0.06] | 0.02 [-0.06;  0.09] | 0.03 [-0.06;  0.11] | 0.00 [-0.05;  0.06] | -0.00 [-0.06;  0.05] | 0.03 [-0.06;  0.11] | Fava bean | . | . | . | . | . | . | . | . | . | . | . |
| Mealworm protein | 0.00 [-0.05;  0.06] | 0.01 [-0.04;  0.06] | 0.02 [-0.04;  0.08] | 0.03 [-0.05;  0.11] | 0.01 [-0.05;  0.06] | -0.00 [-0.05;  0.05] | 0.03 [-0.05;  0.11] | 0.00 [-0.07;  0.08] | Mealworm protein | -0.01 [-0.06; 0.03] | . | . | . | . | . | . | . | . | . |
| Milk protein | -0.01 [-0.04;  0.02] | -0.01 [-0.03;  0.02] | 0.00 [-0.04;  0.05] | 0.02 [-0.05;  0.08] | -0.01 [-0.04;  0.03] | -0.02 [-0.04;  0.01] | 0.01 [-0.05;  0.08] | -0.01 [-0.07;  0.05] | -0.01 [-0.06;  0.03] | Milk protein | -0.02 [-0.06; 0.03] | . | -0.05 [-0.10; 0.01] | . | -0.01 [-0.05; 0.04] | 0.03 [-0.02; 0.09] | . | -0.04 [-0.09; 0.02] | . |
| Mycoprotein | -0.03 [-0.08;  0.03] | -0.03 [-0.08;  0.02] | -0.01 [-0.08;  0.05] | -0.00 [-0.05;  0.04] | -0.03 [-0.08;  0.03] | -0.03 [-0.08;  0.02] | -0.00 [-0.09;  0.08] | -0.03 [-0.10;  0.04] | -0.03 [-0.09;  0.03] | -0.02 [-0.06;  0.02] | Mycoprotein | 0.00 [-0.05; 0.05] | . | -0.01 [-0.05; 0.03] | . | . | 0.01 [-0.04; 0.05] | . | . |
| Mycoprotein concentrate | -0.03 [-0.10;  0.05] | -0.02 [-0.09;  0.05] | -0.01 [-0.09;  0.07] | -0.00 [-0.07;  0.07] | -0.03 [-0.10;  0.05] | -0.03 [-0.10;  0.04] | -0.00 [-0.10;  0.09] | -0.03 [-0.12;  0.06] | -0.03 [-0.11;  0.05] | -0.02 [-0.08;  0.05] | 0.00 [-0.05;  0.05] | Mycoprotein  concentrate | . | . | . | . | . | . | . |
| Native whey | -0.05 [-0.11;  0.01] | -0.04 [-0.10;  0.01] | -0.03 [-0.10;  0.03] | -0.02 [-0.10;  0.06] | -0.04 [-0.10;  0.01] | -0.05 [-0.10;  0.00] | -0.02 [-0.10;  0.06] | -0.05 [-0.12;  0.03] | -0.05 [-0.12;  0.02] | -0.04 [-0.09;  0.02] | -0.02 [-0.08;  0.05] | -0.02 [-0.10;  0.06] | Native whey | . | . | . | . | 0.01 [-0.05; 0.07] | . |
| Pea protein | -0.04 [-0.11;  0.03] | -0.04 [-0.10;  0.03] | -0.03 [-0.10;  0.04] | -0.02 [-0.08;  0.05] | -0.04 [-0.11;  0.03] | -0.05 [-0.11;  0.02] | -0.02 [-0.11;  0.07] | -0.04 [-0.13;  0.04] | -0.05 [-0.12;  0.03] | -0.03 [-0.09;  0.03] | -0.01 [-0.06;  0.03] | -0.02 [-0.08;  0.05] | 0.00 [-0.07;  0.08] | Pea protein | . | . | . | . | . |
| Potato protein | -0.02 [-0.07;  0.04] | -0.01 [-0.06;  0.04] | -0.00 [-0.06;  0.06] | 0.01 [-0.06;  0.09] | -0.01 [-0.07;  0.04] | -0.02 [-0.07;  0.03] | 0.01 [-0.07;  0.09] | -0.02 [-0.09;  0.06] | -0.02 [-0.08;  0.04] | -0.01 [-0.05;  0.04] | 0.01 [-0.05;  0.08] | 0.01 [-0.07;  0.09] | 0.03 [-0.04;  0.10] | 0.03 [-0.05;  0.10] | Potato protein | . | . | . | . |
| Soy | -0.01 [-0.05;  0.03] | -0.00 [-0.03;  0.02] | 0.01 [-0.05;  0.06] | 0.02 [-0.05;  0.09] | -0.01 [-0.04;  0.03] | -0.01 [-0.04;  0.01] | 0.02 [-0.05;  0.09] | -0.01 [-0.07;  0.05] | -0.01 [-0.07;  0.04] | 0.00 [-0.03;  0.03] | 0.02 [-0.03;  0.07] | 0.02 [-0.05;  0.09] | 0.04 [-0.02;  0.10] | 0.03 [-0.03;  0.10] | 0.01 [-0.05;  0.06] | Soy | . | -0.02 [-0.05; 0.01] | . |
| Spirullina | -0.02 [-0.09;  0.05] | -0.02 [-0.09;  0.05] | -0.01 [-0.09;  0.07] | 0.00 [-0.04;  0.05] | -0.02 [-0.09;  0.05] | -0.03 [-0.10;  0.04] | 0.00 [-0.09;  0.10] | -0.02 [-0.11;  0.06] | -0.03 [-0.10;  0.05] | -0.01 [-0.08;  0.05] | 0.01 [-0.04;  0.05] | 0.00 [-0.06;  0.07] | 0.02 [-0.06;  0.10] | 0.02 [-0.04;  0.08] | -0.01 [-0.09;  0.07] | -0.02 [-0.09;  0.06] | Spirullina | . | . |
| Whey | -0.02 [-0.05;  0.02] | -0.01 [-0.03;  0.00] | -0.00 [-0.05;  0.05] | 0.01 [-0.06;  0.08] | -0.02 [-0.04;  0.01] | -0.02 [-0.04; -0.01] | 0.01 [-0.06;  0.07] | -0.02 [-0.07;  0.03] | -0.02 [-0.07;  0.03] | -0.01 [-0.03;  0.02] | 0.01 [-0.04;  0.06] | 0.01 [-0.06;  0.08] | 0.03 [-0.02;  0.08] | 0.03 [-0.04;  0.09] | -0.00 [-0.05;  0.05] | -0.01 [-0.04;  0.02] | 0.01 [-0.06;  0.07] | Whey | . |
| Whole egg protein | -0.03 [-0.09;  0.02] | -0.03 [-0.08;  0.02] | -0.02 [-0.09;  0.05] | -0.01 [-0.09;  0.08] | -0.03 [-0.09;  0.02] | -0.04 [-0.09;  0.01] | -0.01 [-0.05;  0.03] | -0.04 [-0.11;  0.03] | -0.04 [-0.11;  0.03] | -0.02 [-0.08;  0.03] | -0.01 [-0.07;  0.06] | -0.01 [-0.09;  0.08] | 0.01 [-0.06;  0.08] | 0.01 [-0.07;  0.09] | -0.02 [-0.09;  0.05] | -0.03 [-0.08;  0.03] | -0.01 [-0.10;  0.07] | -0.02 [-0.07;  0.03] | Whole egg protein |

REFERENCES

1	Skinner SK, Beals JW, van Vliet S, et al. Muscle Protein Synthetic Responses After Low-dose Protein Ingestion and Resistance Exercise In Older Women. *Medicine & Science in Sports & Exercise* 2018;50(5S):750–51.

2	Reidy PT, Walker DK, Dickinson JM, et al. Protein blend ingestion following resistance exercise promotes human muscle protein synthesis. *The Journal of nutrition* 2013;143(4):410–16. doi:10.3945/jn.112.168021 [published Online First: 23 January 2013].

3	Davies RW, Kozior M, Lynch AE, et al. The Effect of Fava Bean (Vicia faba L.) Protein Ingestion on Myofibrillar Protein Synthesis at Rest and after Resistance Exercise in Healthy, Young Men and Women: A Randomised Control Trial. *Nutrients* 2022;14(18). doi:10.3390/nu14183688 [published Online First: 6 September 2022].

4	van der Heijden I, Monteyne AJ, West S, et al. Plant Protein Blend Ingestion Stimulates Postexercise Myofibrillar Protein Synthesis Rates Equivalently to Whey in Resistance-Trained Adults. *Med Sci Sports Exerc* 2024;56(8):1467–79. doi:10.1249/MSS.0000000000003432 [published Online First: 23 March 2024].

5	Devries MC, McGlory C, Bolster DR, et al. Leucine, Not Total Protein, Content of a Supplement Is the Primary Determinant of Muscle Protein Anabolic Responses in Healthy Older Women. *The Journal of nutrition* 2018;148(7):1088–95.

6	Holwerda AM, Paulussen KJM, Overkamp M, et al. Leucine coingestion augments the muscle protein synthetic response to the ingestion of 15 g of protein following resistance exercise in older men. *Am J Physiol Endocrinol Metab* 2019;317(3):E473-E482. doi:10.1152/ajpendo.00073.2019 [published Online First: 21 May 2019].

7	Hamarsland H, Nordengen AL, Nyvik Aas S, et al. Native whey protein with high levels of leucine results in similar post-exercise muscular anabolic responses as regular whey protein: a randomized controlled trial. *J Int Soc Sports Nutr* 2017;14:43. doi:10.1186/s12970-017-0202-y [published Online First: 21 November 2017].

8	Churchward-Venne TA, Burd NA, Mitchell CJ, et al. Supplementation of a suboptimal protein dose with leucine or essential amino acids: effects on myofibrillar protein synthesis at rest and following resistance exercise in men. *J Physiol* 2012;590(11):2751–65. doi:10.1113/jphysiol.2012.228833 [published Online First: 25 March 2012].

9	Churchward-Venne TA, Breen L, Di Donato DM, et al. Leucine supplementation of a low-protein mixed macronutrient beverage enhances myofibrillar protein synthesis in young men: a double-blind, randomized trial. *Am J Clin Nutr* 2014;99(2):276–86. doi:10.3945/ajcn.113.068775 [published Online First: 27 November 2013].

10	Wilkinson SB, Tarnopolsky MA, Macdonald MJ, et al. Consumption of fluid skim milk promotes greater muscle protein accretion after resistance exercise than does consumption of an isonitrogenous and isoenergetic soy-protein beverage. *Am J Clin Nutr* 2007;85(4):1031–40.

11	Oikawa SY, Kamal MJ, Webb EK, et al. Whey protein but not collagen peptides stimulate acute and longer-term muscle protein synthesis with and without resistance exercise in healthy older women: a randomized controlled trial. *Am J Clin Nutr* 2020;111(3):708–18.

12	Brook MS, Scaife P, Bass JJ, et al. A collagen hydrolysate/milk protein-blend stimulates muscle anabolism equivalently to an isoenergetic milk protein-blend containing a greater quantity of essential amino acids in older men. *Clin Nutr* 2021;40(6):4456–64. doi:10.1016/j.clnu.2021.01.002 [published Online First: 13 January 2021].

13	Yang Y, Churchward-Venne TA, Burd NA, et al. Myofibrillar protein synthesis following ingestion of soy protein isolate at rest and after resistance exercise in elderly men. *Nutr Metab (Lond)* 2012;9(1):57. doi:10.1186/1743-7075-9-57 [published Online First: 14 June 2012].

14	Burd NA, Yang Y, Moore DR, et al. Greater stimulation of myofibrillar protein synthesis with ingestion of whey protein isolate v. micellar casein at rest and after resistance exercise in elderly men. *Br J Nutr* 2012;108(6):958–62. doi:10.1017/S0007114511006271 [published Online First: 31 January 2012].

15	Bukhari SSI, Phillips BE, Wilkinson DJ, et al. Intake of low-dose leucine-rich essential amino acids stimulates muscle anabolism equivalently to bolus whey protein in older women at rest and after exercise. *Am J Physiol Endocrinol Metab* 2015;308(12):E1056-65. doi:10.1152/ajpendo.00481.2014 [published Online First: 31 March 2015].

16	Tang JE, Moore DR, Kujbida GW, et al. Ingestion of whey hydrolysate, casein, or soy protein isolate: effects on mixed muscle protein synthesis at rest and following resistance exercise in young men. *J Appl Physiol (1985)* 2009;107(3):987–92. doi:10.1152/japplphysiol.00076.2009 [published Online First: 9 July 2009].

17	Reitelseder S, Agergaard J, Doessing S, et al. Whey and casein labeled with L-1-13Cleucine and muscle protein synthesis: effect of resistance exercise and protein ingestion. *Am J Physiol Endocrinol Metab* 2011;300(1):E231-42. doi:10.1152/ajpendo.00513.2010 [published Online First: 2 November 2010].

18	Dideriksen KJ, Reitelseder S, Petersen SG, et al. Stimulation of muscle protein synthesis by whey and caseinate ingestion after resistance exercise in elderly individuals. *Scand J Med Sci Sports* 2011;21(6):e372-83. doi:10.1111/j.1600-0838.2011.01318.x [published Online First: 28 April 2011].

19	Areta JL, Burke LM, Ross ML, et al. Timing and distribution of protein ingestion during prolonged recovery from resistance exercise alters myofibrillar protein synthesis. *J Physiol* 2013;591(9):2319–31. doi:10.1113/jphysiol.2012.244897 [published Online First: 4 March 2013].

20	Robinson MJ, Burd NA, Breen L, et al. Dose-dependent responses of myofibrillar protein synthesis with beef ingestion are enhanced with resistance exercise in middle-aged men. *Appl Physiol Nutr Metab* 2013;38(2):120–25. doi:10.1139/apnm-2012-0092 [published Online First: 9 November 2012].

21	Hermans WJ, Fuchs CJ, Nyakayiru J, et al. Acute Quark Ingestion Increases Muscle Protein Synthesis Rates at Rest with a Further Increase after Exercise in Young and Older Adult Males in a Parallel-Group Intervention Trial. *The Journal of nutrition* 2023;153(1):66–75. doi:10.1016/j.tjnut.2022.10.003 [published Online First: 22 December 2022].

22	West S, Monteyne AJ, Whelehan G, et al. Ingestion of mycoprotein, pea protein, and their blend support comparable postexercise myofibrillar protein synthesis rates in resistance-trained individuals. *Am J Physiol Endocrinol Metab* 2023;325(3):E267-E279. doi:10.1152/ajpendo.00166.2023 [published Online First: 2 August 2023].

23	Aussieker T, Hilkens L, Holwerda AM, et al. Collagen Protein Ingestion during Recovery from Exercise Does Not Increase Muscle Connective Protein Synthesis Rates. *Med Sci Sports Exerc* 2023;55(10):1792–802. doi:10.1249/MSS.0000000000003214 [published Online First: 19 May 2023].

24	Hermans WJH, Fuchs CJ, Hendriks FK, et al. Cheese Ingestion Increases Muscle Protein Synthesis Rates Both at Rest and During Recovery from Exercise in Healthy, Young Males: A Randomized Parallel-Group Trial. *The Journal of nutrition* 2022;152(4):1022–30.

25	van der Heijden I, West S, Monteyne AJ, et al. Algae Ingestion Increases Resting and Exercised Myofibrillar Protein Synthesis Rates to a Similar Extent as Mycoprotein in Young Adults. *The Journal of nutrition* 2023;153(12):3406–17. doi:10.1016/j.tjnut.2023.08.035 [published Online First: 15 September 2023].

26	Chan AH, D'Souza RF, Beals JW, et al. The Degree of Aminoacidemia after Dairy Protein Ingestion Does Not Modulate the Postexercise Anabolic Response in Young Men: A Randomized Controlled Trial. *The Journal of nutrition* 2019;149(9):1511–22.

27	Reitelseder S, Dideriksen K, Agergaard J, et al. Even effect of milk protein and carbohydrate intake but no further effect of heavy resistance exercise on myofibrillar protein synthesis in older men. *Eur J Nutr* 2019;58(2):583–95. doi:10.1007/s00394-018-1641-1 [published Online First: 22 February 2018].

28	Holwerda AM, Trommelen J, Kouw IWK, et al. Exercise Plus Presleep Protein Ingestion Increases Overnight Muscle Connective Tissue Protein Synthesis Rates in Healthy Older Men. *Int J Sport Nutr Exerc Metab* 2021;31(3):217–26. doi:10.1123/ijsnem.2020-0222 [published Online First: 14 February 2021].

29	Hermans WJH, Senden JM, Churchward-Venne TA, et al. Insects are a viable protein source for human consumption: from insect protein digestion to postprandial muscle protein synthesis in vivo in humans: a double-blind randomized trial. *Am J Clin Nutr* 2021;114(3):934–44.

30	Monteyne AJ, Coelho MOC, Porter C, et al. Mycoprotein ingestion stimulates protein synthesis rates to a greater extent than milk protein in rested and exercised skeletal muscle of healthy young men: a randomized controlled trial. *Am J Clin Nutr* 2020;112(2):318–33.

31	Trommelen J, Holwerda AM, Senden JM, et al. Casein Ingestion Does Not Increase Muscle Connective Tissue Protein Synthesis Rates. *Med Sci Sports Exerc* 2020;52(9):1983–91.

32	Holm L, Rahbek SK, Farup J, et al. Contraction mode and whey protein intake affect the synthesis rate of intramuscular connective tissue. *Muscle Nerve* 2017;55(1):128–30. doi:10.1002/mus.25398 [published Online First: 26 September 2016].

33	Borack MS, Reidy PT, Husaini SH, et al. Soy-Dairy Protein Blend or Whey Protein Isolate Ingestion Induces Similar Postexercise Muscle Mechanistic Target of Rapamycin Complex 1 Signaling and Protein Synthesis Responses in Older Men. *The Journal of nutrition* 2016;146(12):2468–75. doi:10.3945/jn.116.231159 [published Online First: 26 October 2016].

34	Burd NA, Gorissen SH, van Vliet S, et al. Differences in postprandial protein handling after beef compared with milk ingestion during postexercise recovery: a randomized controlled trial. *Am J Clin Nutr* 2015;102(4):828–36. doi:10.3945/ajcn.114.103184 [published Online First: 9 September 2015].

35	van Vliet S, Shy EL, Abou Sawan S, et al. Consumption of whole eggs promotes greater stimulation of postexercise muscle protein synthesis than consumption of isonitrogenous amounts of egg whites in young men. *Am J Clin Nutr* 2017;106(6):1401–12. doi:10.3945/ajcn.117.159855 [published Online First: 4 October 2017].

36	Moore DR, Robinson MJ, Fry JL, et al. Ingested protein dose response of muscle and albumin protein synthesis after resistance exercise in young men. *Am J Clin Nutr* 2009;89(1):161–68. doi:10.3945/ajcn.2008.26401 [published Online First: 3 December 2008].

37	Yang Y, Breen L, Burd NA, et al. Resistance exercise enhances myofibrillar protein synthesis with graded intakes of whey protein in older men. *Br J Nutr* 2012;108(10):1780–88. doi:10.1017/S0007114511007422 [published Online First: 7 February 2012].

38	Witard OC, Jackman SR, Breen L, et al. Myofibrillar muscle protein synthesis rates subsequent to a meal in response to increasing doses of whey protein at rest and after resistance exercise. *Am J Clin Nutr* 2014;99(1):86–95. doi:10.3945/ajcn.112.055517 [published Online First: 20 November 2013].

39	Macnaughton LS, Wardle SL, Witard OC, et al. The response of muscle protein synthesis following whole-body resistance exercise is greater following 40 g than 20 g of ingested whey protein. *Physiol Rep* 2016;4(15).

40	Holwerda AM, Paulussen KJM, Overkamp M, et al. Dose-Dependent Increases in Whole-Body Net Protein Balance and Dietary Protein-Derived Amino Acid Incorporation into Myofibrillar Protein During Recovery from Resistance Exercise in Older Men. *The Journal of nutrition* 2019;149(2):221–30.

41	Pinckaers PJM, Hendriks FK, Hermans WJH, et al. Potato Protein Ingestion Increases Muscle Protein Synthesis Rates at Rest and during Recovery from Exercise in Humans. *Med Sci Sports Exerc* 2022;54(9):1572–81. doi:10.1249/MSS.0000000000002937 [published Online First: 22 April 2022].

42	West S, Monteyne AJ, Whelehan G, et al. Mycoprotein ingestion within or without its wholefood matrix results in equivalent stimulation of myofibrillar protein synthesis rates in resting and exercised muscle of young men. *Br J Nutr* 2023;130(1):20–32. doi:10.1017/S0007114522003087 [published Online First: 29 September 2022].

43	Mallinson JE, Wardle SL, O'Leary TJ, et al. Protein dose requirements to maximize skeletal muscle protein synthesis after repeated bouts of resistance exercise in young trained women. *Scand J Med Sci Sports* 2023;33(12):2470–81. doi:10.1111/sms.14506 [published Online First: 3 October 2023].

44	Kiera Wilkinson. The effect of a plant based protein source on the skeletal muscle metabolic and functional response to eccentric exercise: (PhD-Thesis) 2023.

45	Paulussen KJ, Barnes TM, Askow AT, et al. Underpinning the Food Matrix Regulation of Postexercise Myofibrillar Protein Synthesis by Comparing Salmon Ingestion With the Sum of Its Isolated Nutrients in Healthy Young Adults. *The Journal of nutrition* 2023;153(5):1359–72. doi:10.1016/j.tjnut.2023.02.037 [published Online First: 2 March 2023].

46	Aussieker T, Kaiser J, Hendriks FK, et al. Effects Of Collagen Peptide Versus Free Amino Acid Ingestion On Muscle Connective Protein Synthesis Rates. *Medicine & Science in Sports & Exercise* 2024;56(10S):1123.

47	McKenna CF, Askow AT, Paulussen KJM, et al. Postabsorptive and postprandial myofibrillar protein synthesis rates at rest and after resistance exercise in women with postmenopause. *J Appl Physiol (1985)* 2024;136(6):1388–99. doi:10.1152/japplphysiol.00886.2023 [published Online First: 22 February 2024].

48	Pennings B, Koopman R, Beelen M, et al. Exercising before protein intake allows for greater use of dietary protein-derived amino acids for de novo muscle protein synthesis in both young and elderly men. *Am J Clin Nutr* 2011;93(2):322–31. doi:10.3945/ajcn.2010.29649 [published Online First: 17 November 2010].

49	Nishimura Y, Jensen M, Bülow J, et al. Co-ingestion of cluster dextrin carbohydrate does not increase exogenous protein-derived amino acid release or myofibrillar protein synthesis following a whole-body resistance exercise in moderately trained younger males: a double-blinded randomized controlled crossover trial. *Eur J Nutr* 2022;61(5):2475–91. doi:10.1007/s00394-021-02782-y [published Online First: 19 February 2022].

50	Koopman R, Verdijk LB, Beelen M, et al. Co-ingestion of leucine with protein does not further augment post-exercise muscle protein synthesis rates in elderly men. *Br J Nutr* 2008;99(3):571–80. doi:10.1017/S0007114507812013 [published Online First: 13 August 2007].

51	Rahbek SK, Farup J, Møller AB, et al. Effects of divergent resistance exercise contraction mode and dietary supplementation type on anabolic signalling, muscle protein synthesis and muscle hypertrophy. *Amino Acids* 2014;46(10):2377–92. doi:10.1007/s00726-014-1792-1 [published Online First: 9 July 2014].
