# Supplementary material for: Impact of different protein sources on acute muscle protein synthesis following resistance training: a systematic review with standard and dose–response network meta-analyses
Source: J Int Soc Sports Nutr. 2026 Oct 12;23(1):2740224. doi: 10.1080/15502783.2026.2740224 (PMC13660545; doi:10.1080/15502783.2026.2740224)
Supplement: Supplementary file 1 [file RSSN_A_2740224_SM5642.zip › bischof_FigureS1.pdf]

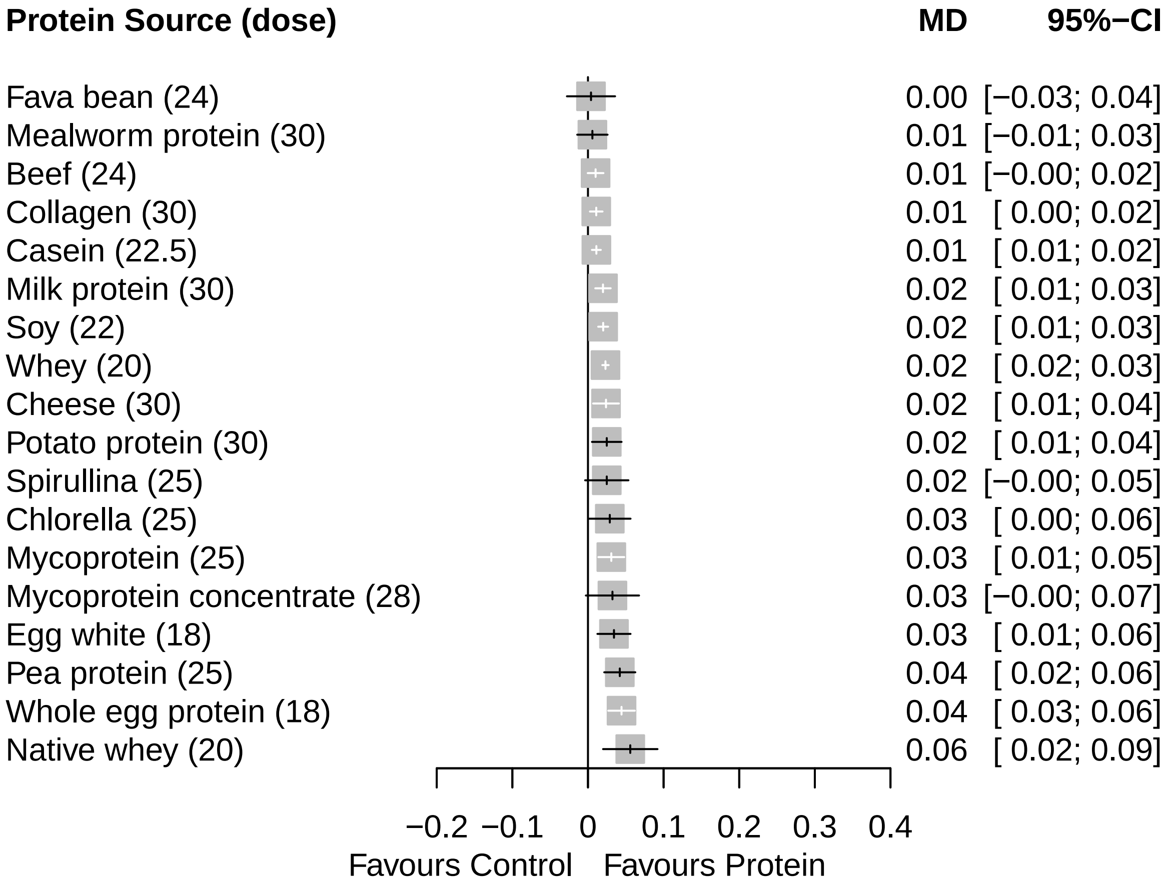

Protein Source (dose)
MD
95%−CI
Fava bean (24) 0.00 [−0.03; 0.04]
Mealworm protein (30) 0.01 [−0.01; 0.03]
Beef (24) 0.01 [−0.00; 0.02]
Collagen (30) 0.01 [0.00; 0.02]
Casein (22.5) 0.01 [0.01; 0.02]
Milk protein (30) 0.02 [0.01; 0.03]
Soy (22) 0.02 [0.01; 0.03]
Whey (20) 0.02 [0.02; 0.03]
Cheese (30) 0.02 [0.01; 0.04]
Potato protein (30) 0.02 [0.01; 0.04]
Spirullina (25) 0.02 [−0.00; 0.05]
Chlorella (25) 0.03 [0.00; 0.06]
Mycoprotein (25) 0.03 [0.01; 0.05]
Mycoprotein concentrate (28) 0.03 [−0.00; 0.07]
Egg white (18) 0.03 [0.01; 0.06]
Pea protein (25) 0.04 [0.02; 0.06]
Whole egg protein (18) 0.04 [0.03; 0.06]
Native whey (20) 0.06 [0.02; 0.09]
−0.2 −0.1 0 0.1 0.2 0.3 0.4
Favours Control
Favours Protein
