# Supplementary material for: Impact of different protein sources on acute muscle protein synthesis following resistance training: a systematic review with standard and dose–response network meta-analyses
Source: J Int Soc Sports Nutr. 2026 Oct 12;23(1):2740224. doi: 10.1080/15502783.2026.2740224 (PMC13660545; doi:10.1080/15502783.2026.2740224)
Supplement: Supplementary file 1 [file RSSN_A_2740224_SM5642.zip › bischof_FigureS2.pdf]

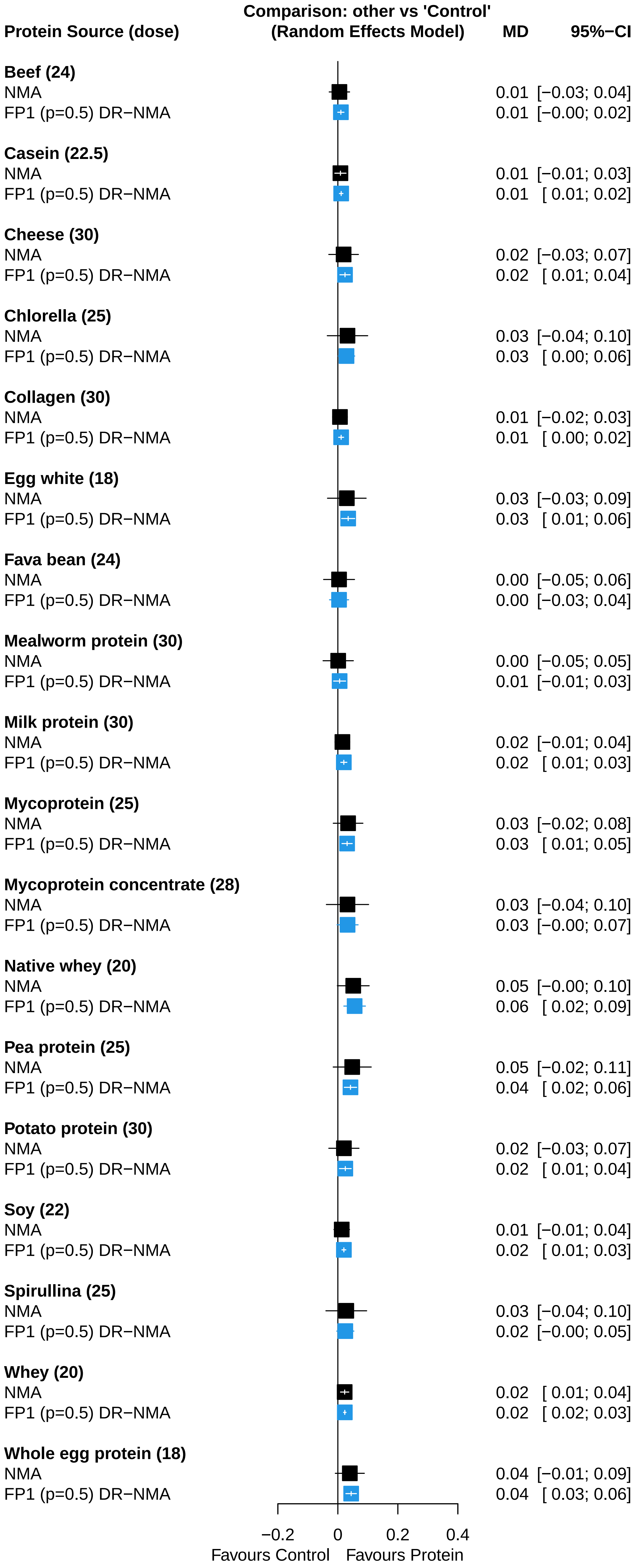

Comparison: other vs 'Control'
(Random Effects Model)
Protein Source (dose)
MD
95%−CI
Beef (24)
NMA 0.01 [−0.03; 0.04]
FP1 (p=0.5) DR−NMA 0.01 [−0.00; 0.02]
Casein (22.5)
NMA 0.01 [−0.01; 0.03]
FP1 (p=0.5) DR−NMA 0.01 [0.01; 0.02]
Cheese (30)
NMA 0.02 [−0.03; 0.07]
FP1 (p=0.5) DR−NMA 0.02 [0.01; 0.04]
Chlorella (25)
NMA 0.03 [−0.04; 0.10]
FP1 (p=0.5) DR−NMA 0.03 [0.00; 0.06]
Collagen (30)
NMA 0.01 [−0.02; 0.03]
FP1 (p=0.5) DR−NMA 0.01 [0.00; 0.02]
Egg white (18)
NMA 0.03 [−0.03; 0.09]
FP1 (p=0.5) DR−NMA 0.03 [0.01; 0.06]
Fava bean (24)
NMA 0.00 [−0.05; 0.06]
FP1 (p=0.5) DR−NMA 0.00 [−0.03; 0.04]
Mealworm protein (30)
NMA 0.00 [−0.05; 0.05]
FP1 (p=0.5) DR−NMA 0.01 [−0.01; 0.03]
Milk protein (30)
NMA 0.02 [−0.01; 0.04]
FP1 (p=0.5) DR−NMA 0.02 [0.01; 0.03]
Mycoprotein (25)
NMA 0.03 [−0.02; 0.08]
FP1 (p=0.5) DR−NMA 0.03 [0.01; 0.05]
Mycoprotein concentrate (28)
NMA 0.03 [−0.04; 0.10]
FP1 (p=0.5) DR−NMA 0.03 [−0.00; 0.07]
Native whey (20)
NMA 0.05 [−0.00; 0.10]
FP1 (p=0.5) DR−NMA 0.06 [0.02; 0.09]
Pea protein (25)
NMA 0.05 [−0.02; 0.11]
FP1 (p=0.5) DR−NMA 0.04 [0.02; 0.06]
Potato protein (30)
NMA 0.02 [−0.03; 0.07]
FP1 (p=0.5) DR−NMA 0.02 [0.01; 0.04]
Soy (22)
NMA 0.01 [−0.01; 0.04]
FP1 (p=0.5) DR−NMA 0.02 [0.01; 0.03]
Spirullina (25)
NMA 0.03 [−0.04; 0.10]
FP1 (p=0.5) DR−NMA 0.02 [−0.00; 0.05]
Whey (20)
NMA 0.02 [0.01; 0.04]
FP1 (p=0.5) DR−NMA 0.02 [0.02; 0.03]
Whole egg protein (18)
NMA 0.04 [−0.01; 0.09]
FP1 (p=0.5) DR−NMA 0.04 [0.03; 0.06]
−0.2
0
0.2
0.4
Favours Control
Favours Protein
