# Supplementary material for: Impact of different protein sources on acute muscle protein synthesis following resistance training: a systematic review with standard and dose–response network meta-analyses
Source: J Int Soc Sports Nutr. 2026 Oct 12;23(1):2740224. doi: 10.1080/15502783.2026.2740224 (PMC13660545; doi:10.1080/15502783.2026.2740224)
Supplement: Supplementary file 1 [file RSSN_A_2740224_SM5642.zip › bischof_FigureS3.pdf]

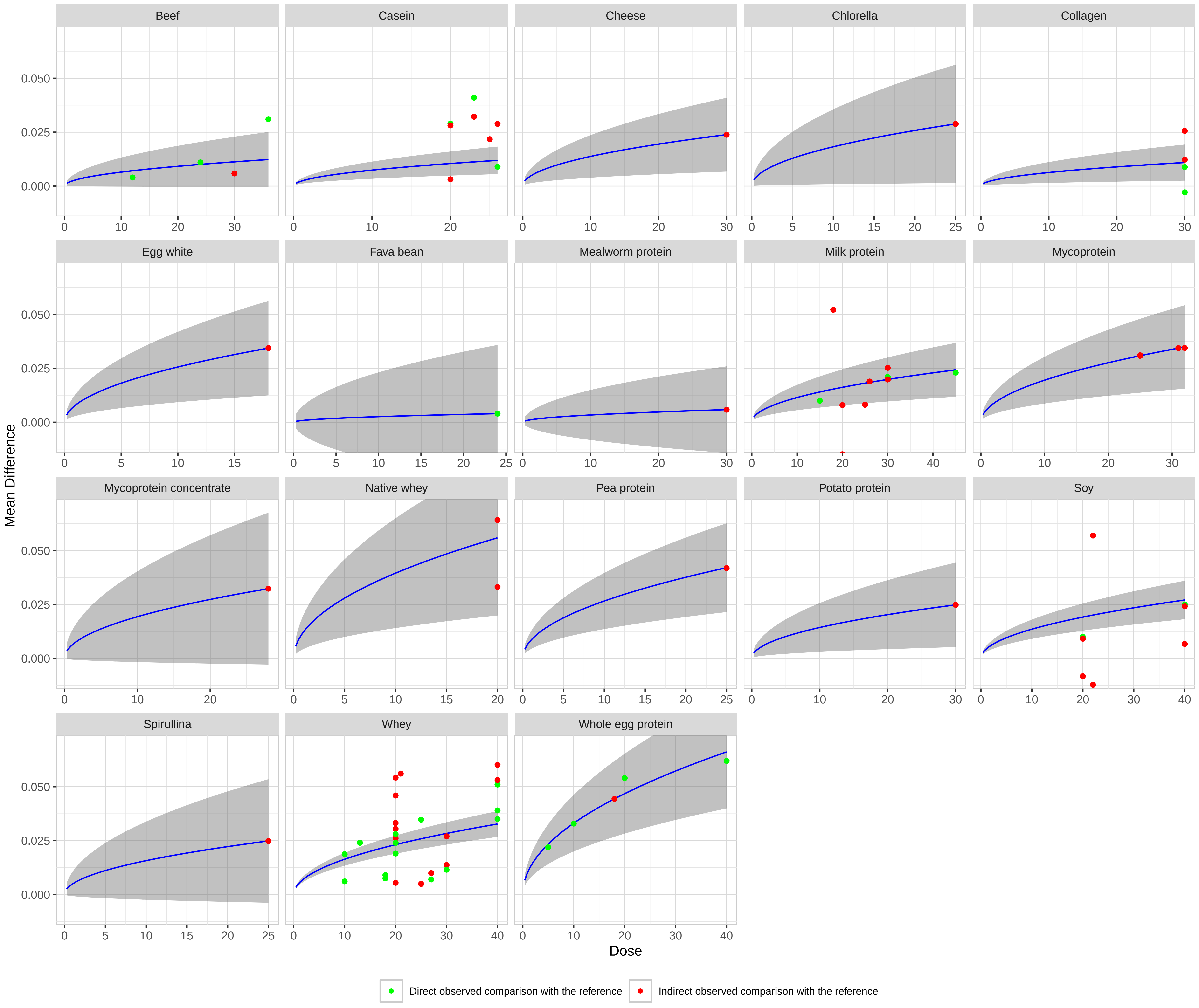

Beef
Casein
Cheese
Chlorella
Collagen
Egg white
Fava bean
Mealworm protein
Milk protein
Mycoprotein
Mycoprotein concentrate
Native whey
Pea protein
Potato protein
Soy
Spirullina
Whey
Whole egg protein
Mean Difference
Dose
0.050
0.025
0.000
Direct observed comparison with the reference
Indirect observed comparison with the reference
