# Supplementary material for: Impact of different protein sources on acute muscle protein synthesis following resistance training: a systematic review with standard and dose–response network meta-analyses
Source: J Int Soc Sports Nutr. 2026 Oct 12;23(1):2740224. doi: 10.1080/15502783.2026.2740224 (PMC13660545; doi:10.1080/15502783.2026.2740224)

Comparison	Number of Studies	Direct Evidence	I2	MD	95%−CI
**Beef vs Control**					
Direct estimate	1	0.57		0.01	[−0.04; 0.05]
Indirect estimate				−0.00	[−0.05; 0.05]
Network estimate				0.01	[−0.03; 0.04]
**Beef vs Milk protein**					
Direct estimate	1	0.58		−0.01	[−0.06; 0.03]
Indirect estimate				−0.00	[−0.06; 0.05]
Network estimate				−0.01	[−0.04; 0.02]
**Casein vs Control**					
Direct estimate	3	0.55	92.6%	0.03	[0.00; 0.05]
Indirect estimate				−0.01	[−0.04; 0.02]
Network estimate				0.01	[−0.01; 0.03]
**Casein vs Milk protein**					
Direct estimate	1	0.32		0.00	[−0.04; 0.05]
Indirect estimate				−0.01	[−0.04; 0.02]
Network estimate				−0.01	[−0.03; 0.02]
**Casein vs Soy**					
Direct estimate	1	0.41		−0.05	[−0.09; −0.00]
Indirect estimate				0.02	[−0.01; 0.06]
Network estimate				−0.00	[−0.03; 0.02]
**Casein vs Whey**					
Direct estimate	5	0.81	97.4%	−0.02	[−0.04; 0.00]
Indirect estimate				0.00	[−0.04; 0.04]
Network estimate				−0.01	[−0.03; 0.00]
**Collagen vs Control**					
Direct estimate	2	0.65	44.3%	0.00	[−0.03; 0.03]
Indirect estimate				0.01	[−0.03; 0.06]
Network estimate				0.01	[−0.02; 0.03]
**Collagen vs Whey**					
Direct estimate	2	0.66	72.6%	−0.01	[−0.04; 0.02]
Indirect estimate				−0.03	[−0.07; 0.01]
Network estimate				−0.02	[−0.04; 0.01]
**Milk protein vs Control**					
Direct estimate	1	0.31		0.02	[−0.03; 0.06]
Indirect estimate				0.01	[−0.02; 0.04]
Network estimate				0.02	[−0.01; 0.04]
**Pea protein vs Control**					
Direct estimate	1	0.07		0.14	[−0.10; 0.38]
Indirect estimate				0.04	[−0.02; 0.11]
Network estimate				0.05	[−0.02; 0.11]
**Soy vs Control**					
Direct estimate	1	0.38		0.02	[−0.02; 0.06]
Indirect estimate				0.01	[−0.02; 0.04]
Network estimate				0.01	[−0.01; 0.04]
**Whey vs Control**					
Direct estimate	10	0.85	75.0%	0.02	[0.00; 0.03]
Indirect estimate				0.05	[0.02; 0.09]
Network estimate				0.02	[0.01; 0.04]
**Milk protein vs Mycoprotein**					
Direct estimate	1	0.97		−0.02	[−0.06; 0.03]
Indirect estimate				−0.11	[−0.36; 0.13]
Network estimate				−0.02	[−0.06; 0.02]
**Milk protein vs Native whey**					
Direct estimate	1	0.87		−0.05	[−0.10; 0.01]
Indirect estimate				0.05	[−0.10; 0.19]
Network estimate				−0.04	[−0.09; 0.02]
**Milk protein vs Soy**					
Direct estimate	1	0.30		0.03	[−0.02; 0.09]
Indirect estimate				−0.01	[−0.05; 0.03]
Network estimate				0.00	[−0.03; 0.03]
**Milk protein vs Whey**					
Direct estimate	1	0.20		−0.04	[−0.09; 0.02]
Indirect estimate				0.00	[−0.03; 0.03]
Network estimate				−0.01	[−0.03; 0.02]
**Mycoprotein vs Pea protein**					
Direct estimate	1	0.97		−0.01	[−0.05; 0.03]
Indirect estimate				−0.11	[−0.35; 0.14]
Network estimate				−0.01	[−0.06; 0.03]
**Native whey vs Whey**					
Direct estimate	1	0.75		0.01	[−0.05; 0.07]
Indirect estimate				0.08	[−0.02; 0.19]
Network estimate				0.03	[−0.02; 0.08]
**Soy vs Whey**					
Direct estimate	2	0.66	85.1%	−0.02	[−0.05; 0.01]
Indirect estimate				0.01	[−0.03; 0.06]
Network estimate				−0.01	[−0.04; 0.02]

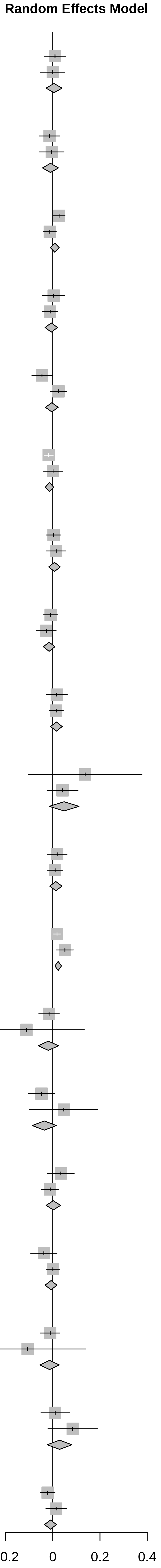

Supplement: Supplementary file 1 [file RSSN_A_2740224_SM5642.zip › bischof_figureS4.pdf]
