# Supplementary material for: Impact of different protein sources on acute muscle protein synthesis following resistance training: a systematic review with standard and dose–response network meta-analyses
Source: J Int Soc Sports Nutr. 2026 Oct 12;23(1):2740224. doi: 10.1080/15502783.2026.2740224 (PMC13660545; doi:10.1080/15502783.2026.2740224)
Supplement: Supplementary file 1 [file RSSN_A_2740224_SM5642.zip › bischof_figureS5.pdf]

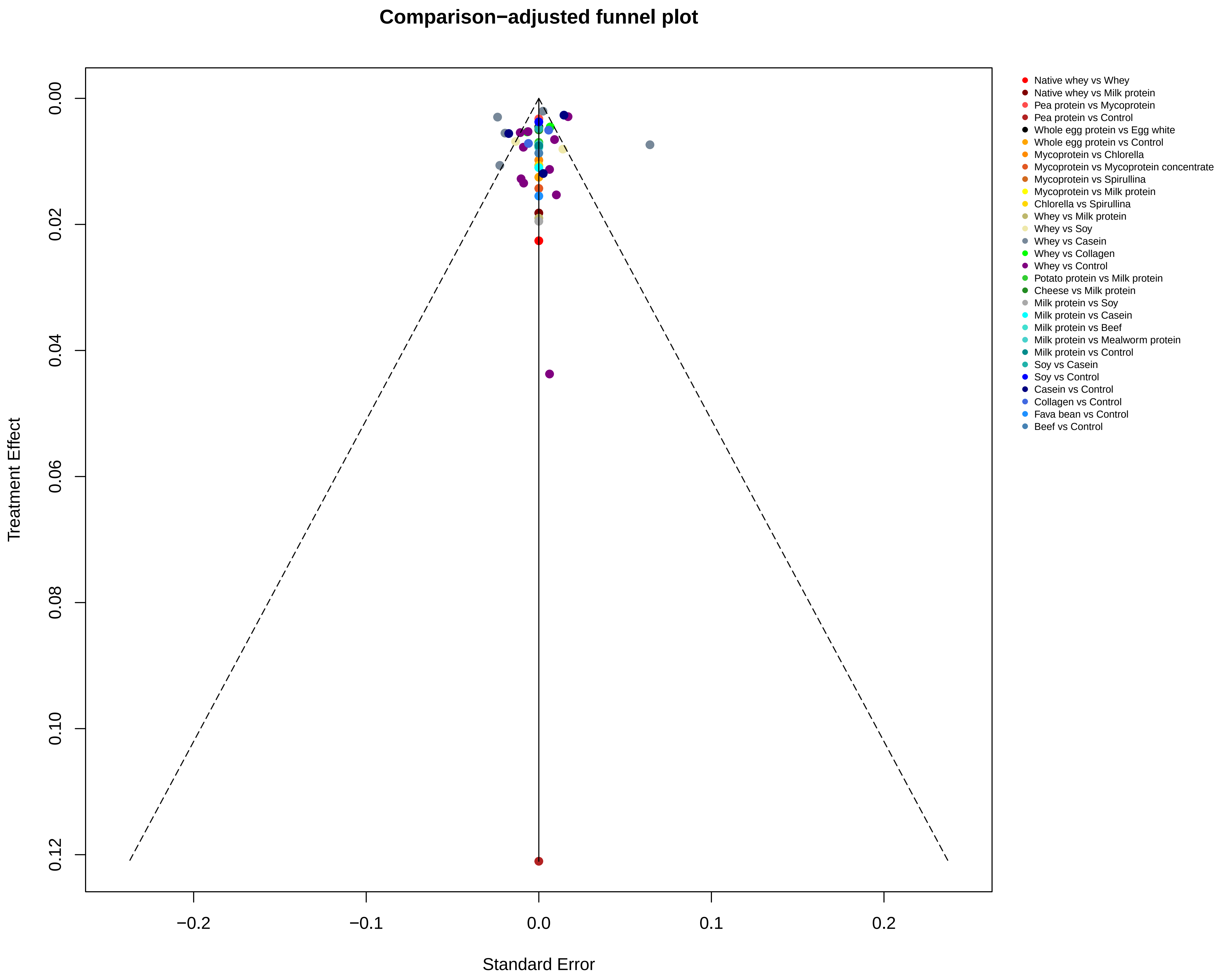
Comparison−adjusted funnel plot
Treatment Effect
0.00
0.02
0.04
0.06
0.08
0.10
0.12
−0.2
−0.1
0.0
0.1
0.2
Standard Error
Native whey vs Whey
Native whey vs Milk protein
Pea protein vs Mycoprotein
Pea protein vs Control
Whole egg protein vs Egg white
Whole egg protein vs Control
Mycoprotein vs Chlorella
Mycoprotein vs Mycoprotein concentrate
Mycoprotein vs Spirullina
Mycoprotein vs Milk protein
Chlorella vs Spirullina
Whey vs Milk protein
Whey vs Soy
Whey vs Casein
Whey vs Collagen
Whey vs Control
Potato protein vs Milk protein
Cheese vs Milk protein
Milk protein vs Soy
Milk protein vs Casein
Milk protein vs Beef
Milk protein vs Mealworm protein
Milk protein vs Control
Soy vs Casein
Soy vs Control
Casein vs Control
Collagen vs Control
Fava bean vs Control
Beef vs Control
