# Supplementary material for: Impact of different protein sources on acute muscle protein synthesis following resistance training: a systematic review with standard and dose–response network meta-analyses
Source: J Int Soc Sports Nutr. 2026 Oct 12;23(1):2740224. doi: 10.1080/15502783.2026.2740224 (PMC13660545; doi:10.1080/15502783.2026.2740224)
Supplement: Supplementary file 1 [file RSSN_A_2740224_SM5642.zip › bischof_figureS6.pdf]

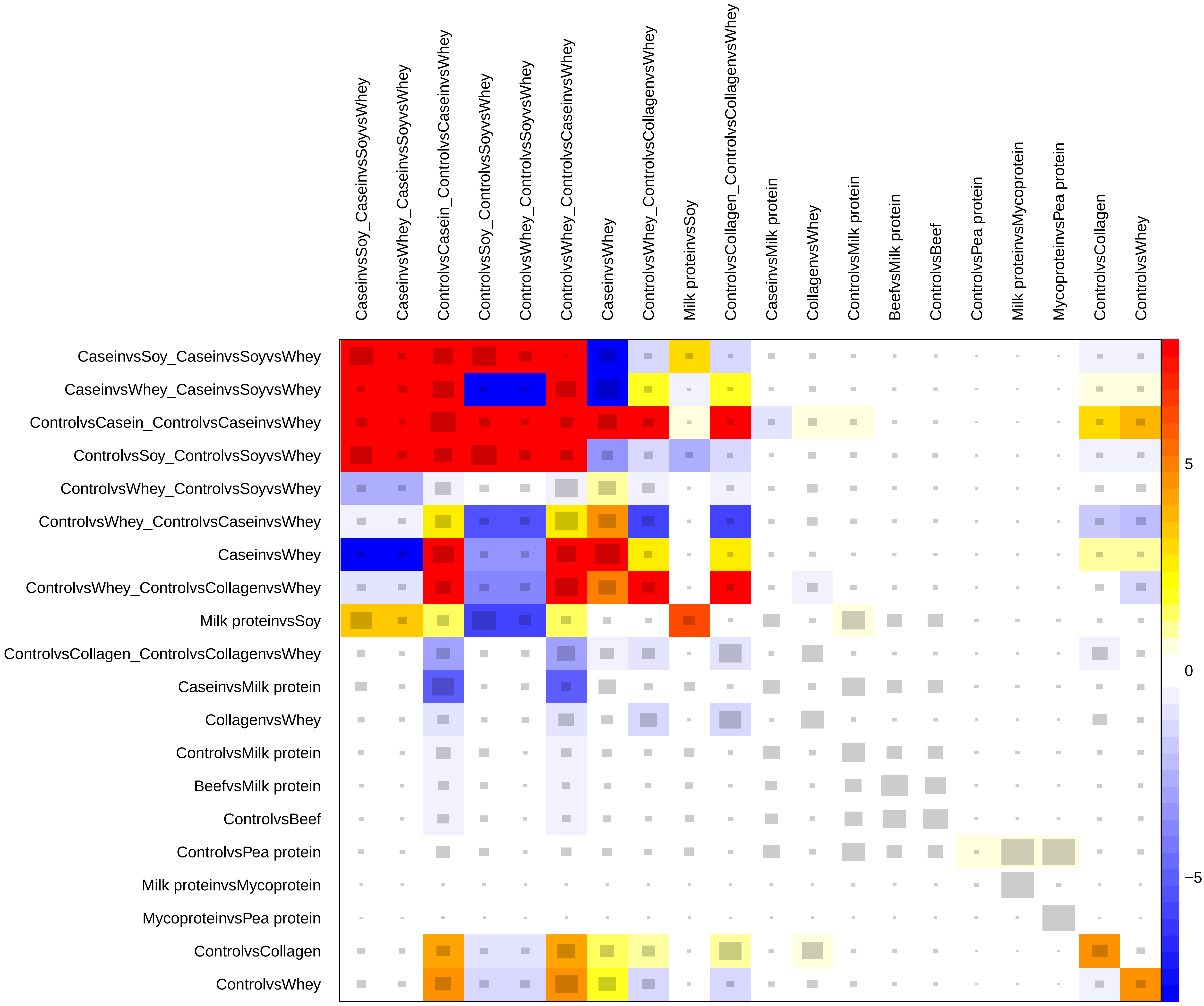
CaseinvsSoy_CaseinvsSoyvsWhey
CaseinvsWhey_CaseinvsSoyvsWhey
ControlvsCasein_ControlvsCaseinvsWhey
ControlvsSoy_ControlvsSoyvsWhey
ControlvsWhey_ControlvsSoyvsWhey
ControlvsWhey_ControlvsCaseinvsWhey
CaseinvsWhey
ControlvsWhey_ControlvsCollagenvsWhey
Milk proteinvsSoy
ControlvsCollagen_ControlvsCollagenvsWhey
CaseinvsMilk protein
CollagenvsWhey
ControlvsMilk protein
BeefvsMilk protein
ControlvsBeef
ControlvsPea protein
Milk proteinvsMycoprotein
MycoproteinvsPea protein
ControlvsCollagen
ControlvsWhey
5
0
−5
