# Supplementary material for: Impact of different protein sources on acute muscle protein synthesis following resistance training: a systematic review with standard and dose–response network meta-analyses
Source: J Int Soc Sports Nutr. 2026 Oct 12;23(1):2740224. doi: 10.1080/15502783.2026.2740224 (PMC13660545; doi:10.1080/15502783.2026.2740224)
Supplement: Supplementary file 1 [file RSSN_A_2740224_SM5642.zip › bischof_figureS7.pdf]

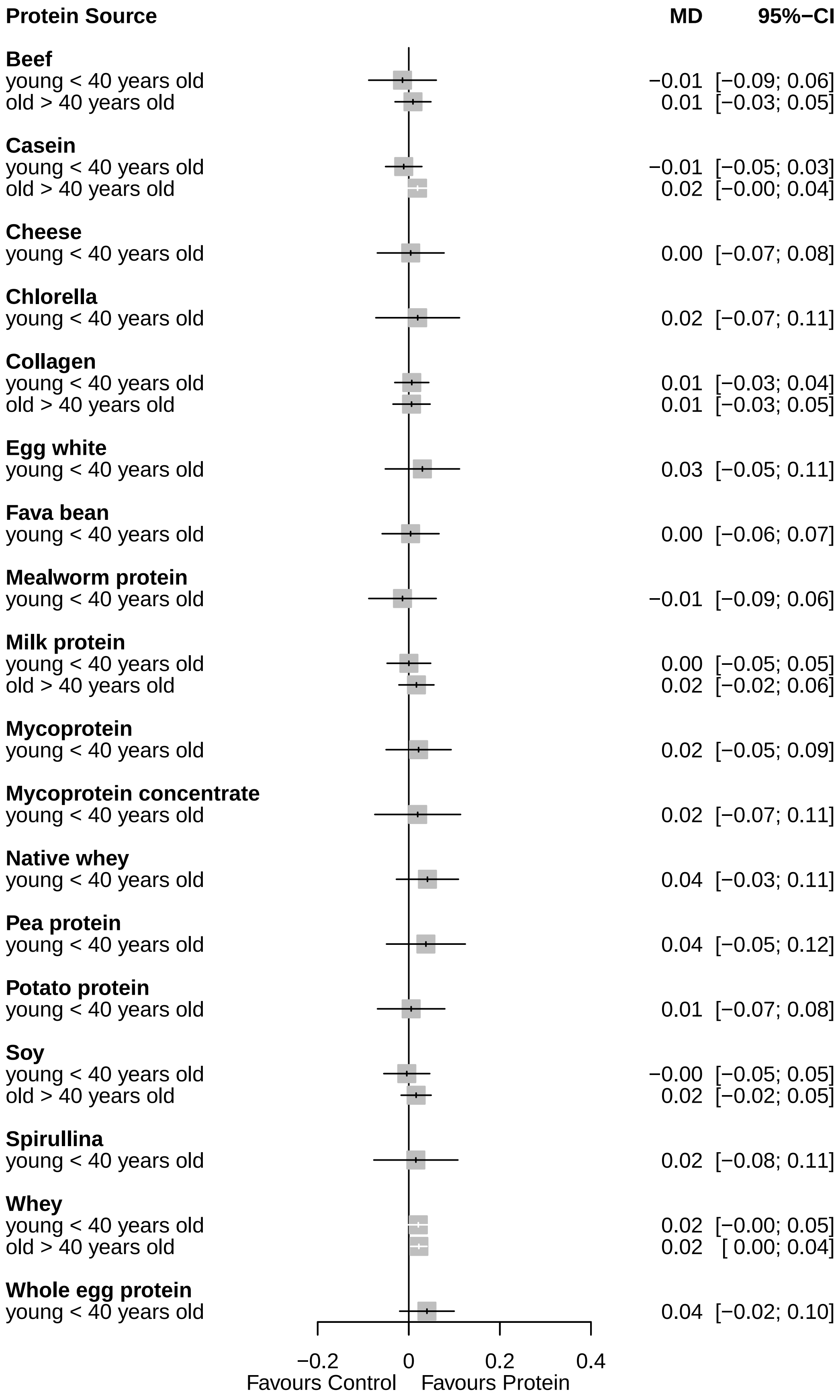
Protein Source
MD
95%−CI
Beef
young < 40 years old −0.01 [−0.09; 0.06]
old > 40 years old 0.01 [−0.03; 0.05]
Casein
young < 40 years old −0.01 [−0.05; 0.03]
old > 40 years old 0.02 [−0.00; 0.04]
Cheese
young < 40 years old 0.00 [−0.07; 0.08]
Chlorella
young < 40 years old 0.02 [−0.07; 0.11]
Collagen
young < 40 years old 0.01 [−0.03; 0.04]
old > 40 years old 0.01 [−0.03; 0.05]
Egg white
young < 40 years old 0.03 [−0.05; 0.11]
Fava bean
young < 40 years old 0.00 [−0.06; 0.07]
Mealworm protein
young < 40 years old −0.01 [−0.09; 0.06]
Milk protein
young < 40 years old 0.00 [−0.05; 0.05]
old > 40 years old 0.02 [−0.02; 0.06]
Mycoprotein
young < 40 years old 0.02 [−0.05; 0.09]
Mycoprotein concentrate
young < 40 years old 0.02 [−0.07; 0.11]
Native whey
young < 40 years old 0.04 [−0.03; 0.11]
Pea protein
young < 40 years old 0.04 [−0.05; 0.12]
Potato protein
young < 40 years old 0.01 [−0.07; 0.08]
Soy
young < 40 years old −0.00 [−0.05; 0.05]
old > 40 years old 0.02 [−0.02; 0.05]
Spirullina
young < 40 years old 0.02 [−0.08; 0.11]
Whey
young < 40 years old 0.02 [−0.00; 0.05]
old > 40 years old 0.02 [ 0.00; 0.04]
Whole egg protein
young < 40 years old 0.04 [−0.02; 0.10]
−0.2
0
0.2
0.4
Favours Control
Favours Protein
