# Supplementary material for: Impact of different protein sources on acute muscle protein synthesis following resistance training: a systematic review with standard and dose–response network meta-analyses
Source: J Int Soc Sports Nutr. 2026 Oct 12;23(1):2740224. doi: 10.1080/15502783.2026.2740224 (PMC13660545; doi:10.1080/15502783.2026.2740224)
Supplement: Supplementary file 1 [file RSSN_A_2740224_SM5642.zip › bischof_figureS8.pdf]

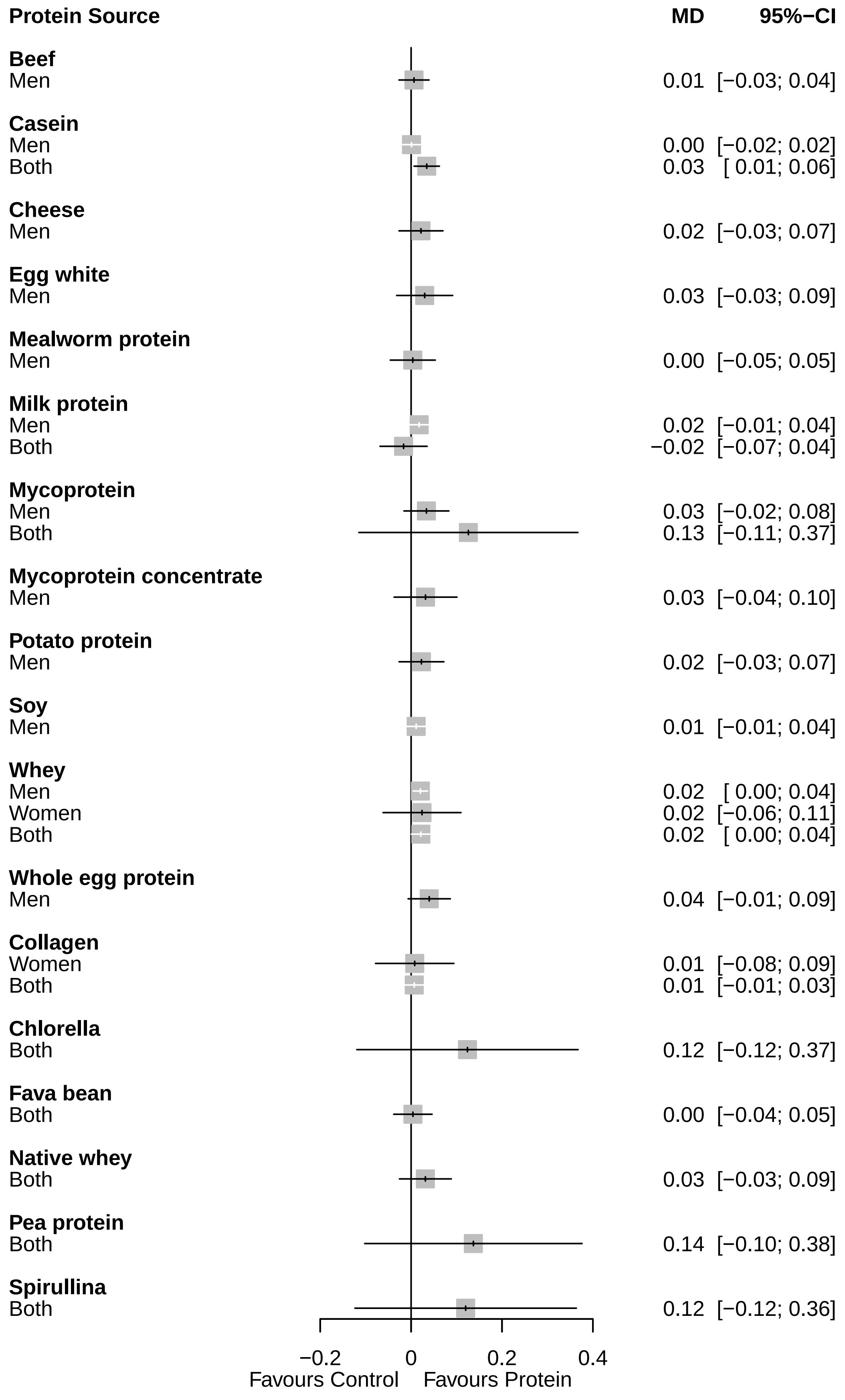
Protein Source
MD
95%−CI
Beef
Men 0.01 [−0.03; 0.04]
Casein
Men 0.00 [−0.02; 0.02]
Both 0.03 [0.01; 0.06]
Cheese
Men 0.02 [−0.03; 0.07]
Egg white
Men 0.03 [−0.03; 0.09]
Mealworm protein
Men 0.00 [−0.05; 0.05]
Milk protein
Men 0.02 [−0.01; 0.04]
Both −0.02 [−0.07; 0.04]
Mycoprotein
Men 0.03 [−0.02; 0.08]
Both 0.13 [−0.11; 0.37]
Mycoprotein concentrate
Men 0.03 [−0.04; 0.10]
Potato protein
Men 0.02 [−0.03; 0.07]
Soy
Men 0.01 [−0.01; 0.04]
Whey
Men 0.02 [0.00; 0.04]
Women 0.02 [−0.06; 0.11]
Both 0.02 [0.00; 0.04]
Whole egg protein
Men 0.04 [−0.01; 0.09]
Collagen
Women 0.01 [−0.08; 0.09]
Both 0.01 [−0.01; 0.03]
Chlorella
Both 0.12 [−0.12; 0.37]
Fava bean
Both 0.00 [−0.04; 0.05]
Native whey
Both 0.03 [−0.03; 0.09]
Pea protein
Both 0.14 [−0.10; 0.38]
Spirullina
Both 0.12 [−0.12; 0.36]
−0.2
0
0.2
0.4
Favours Control
Favours Protein
