# Supplementary material for: Impact of different protein sources on acute muscle protein synthesis following resistance training: a systematic review with standard and dose–response network meta-analyses
Source: J Int Soc Sports Nutr. 2026 Oct 12;23(1):2740224. doi: 10.1080/15502783.2026.2740224 (PMC13660545; doi:10.1080/15502783.2026.2740224)

Intervention	MD	95%−CI
**Control**		
collagenous protein	−0.01	[−0.04; 0.02]
myofibrillar protein	−0.02	[−0.03; −0.01]
myofibrillar+collagenous protein	−0.02	[−0.04; −0.00]
**Beef**		
myofibrillar protein	−0.01	[−0.04; 0.01]
**Casein**		
myofibrillar protein	−0.01	[−0.02; 0.01]
myofibrillar+collagenous protein	0.01	[−0.01; 0.04]
whole protein synthesis (collagen+myofibrillar+sarcoplasmic+mitochondrial)	−0.09	[−0.11; −0.06]
**Chlorella**		
myofibrillar protein	0.11	[−0.14; 0.35]
**Collagen**		
myofibrillar protein	−0.02	[−0.04; 0.01]
myofibrillar+collagenous protein	−0.01	[−0.04; 0.01]
**Fava bean**		
myofibrillar protein	−0.01	[−0.05; 0.02]
**Milk protein**		
myofibrillar protein	−0.00	[−0.02; 0.02]
whole protein synthesis (collagen+myofibrillar+sarcoplasmic+mitochondrial)	−0.02	[−0.06; 0.01]
**Mycoprotein**		
myofibrillar protein	0.11	[−0.13; 0.35]
whole protein synthesis (collagen+myofibrillar+sarcoplasmic+mitochondrial)	−0.01	[−0.05; 0.04]
**Mycoprotein concentrate**		
myofibrillar protein	0.11	[−0.14; 0.35]
**Pea protein**		
myofibrillar protein	0.12	[−0.12; 0.36]
**Soy**		
myofibrillar protein	−0.00	[−0.02; 0.02]
whole protein synthesis (collagen+myofibrillar+sarcoplasmic+mitochondrial)	−0.04	[−0.07; −0.02]
**Spirullina**		
myofibrillar protein	0.10	[−0.14; 0.34]
**Cheese**		
whole protein synthesis (collagen+myofibrillar+sarcoplasmic+mitochondrial)	−0.02	[−0.06; 0.02]
**Mealworm protein**		
whole protein synthesis (collagen+myofibrillar+sarcoplasmic+mitochondrial)	−0.04	[−0.08; 0.01]
**Native whey**		
whole protein synthesis (collagen+myofibrillar+sarcoplasmic+mitochondrial)	0.02	[−0.03; 0.07]
**Potato protein**		
whole protein synthesis (collagen+myofibrillar+sarcoplasmic+mitochondrial)	−0.02	[−0.06; 0.03]

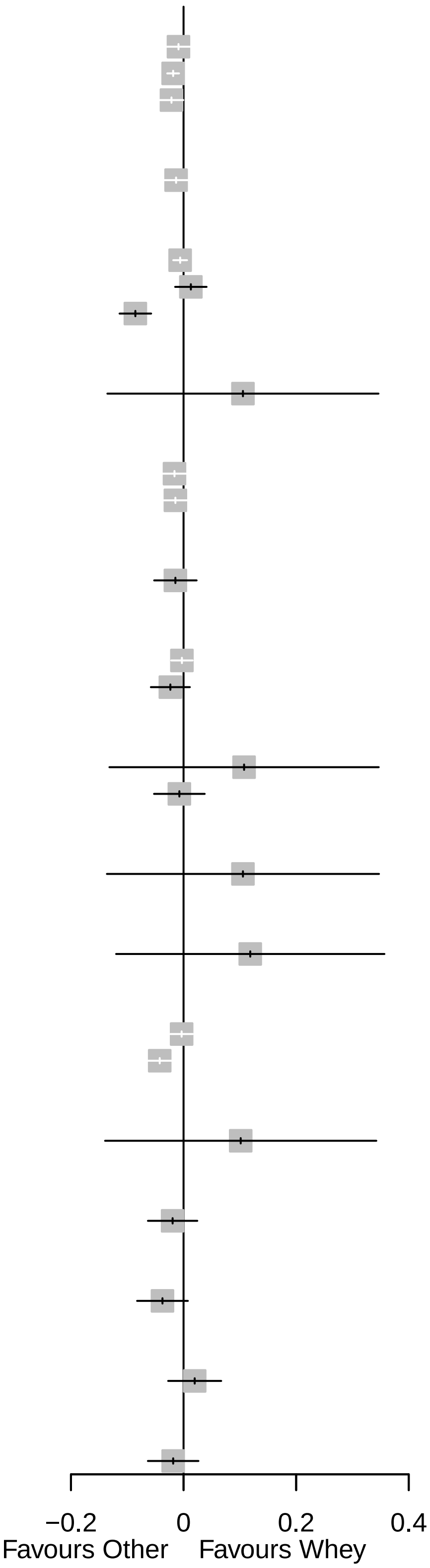

Supplement: Supplementary file 1 [file RSSN_A_2740224_SM5642.zip › bischof_figureS9.pdf]
